# Topological data analysis captures horizontal gene transfer in antimicrobial resistance gene families among clinically relevant bacteria

**DOI:** 10.3389/fmicb.2025.1461293

**Published:** 2025-05-07

**Authors:** Shaday Guerrero-Flores, Haydeé Contreras-Peruyero, José María Ibarra-Rodríguez, José Abel Lovaco-Flores, Francisco Santiago Nieto-de la Rosa, Fernando Fontove-Herrera, Nelly Sélem-Mojica

**Affiliations:** ^1^Centro de Ciencias Matemáticas, Universidad Nacional Autónoma de México, Morelia, Mexico; ^2^C3-Idea, Guanajuato, León, Mexico; ^3^Department of Genetic Engineering, Center for Research and Advanced Studies of the National Polytechnic Institute (CINVESTAV), Irapuato, Mexico

**Keywords:** antimicrobial resistance (AMR), persistence barcode, horizontal gene transfer (HGT), topological data analysis, persistent homology

## Abstract

Antibiotic resistance, projected to cause 10 million deaths annually by 2050, remains a critical health threat. Hospitals drive multidrug resistance via horizontal gene transfer. The 2023 Critical Assessment of Massive Data Analysis challenge presents resistance markers from 146 Johns Hopkins bacterial isolates, aiming to analyze resistomes without metadata or genomic sequences. Persistent homology, a topological data analysis method, effectively captures processes beyond vertical inheritance. A 1-hole is a topological feature representing a loop or gap in the data, where relationships form a circular structure rather than a linear one. Unlike vertical inheritance, which lacks topological 1-holes, horizontal gene transfer generates distinct patterns. Since antimicrobial resistance genes often spread via horizontal gene transfer, we simulated vertical and horizontal inheritance in bacterial resistomes. The number of 1-holes from simulations and a documented horizontal gene transfer case was analyzed using persistence barcodes. In a simulated population of binary sequences, we observed that, on average, two 1-holes form for every three genomes undergoing horizontal gene transfer. Using a presence-absence gene table, we confirmed the existence of 1-holes in a documented case of horizontal gene transfer between two bacterial genera in a Pittsburgh hospital. Notably, the Critical Assessment of Massive Data Analysis resistomes of *Klebsiella* and *Escherichia* exhibit 1-holes, while *Enterobacter* shows none. Lastly, we provide a mathematical example of a non-tree-like space that contains no 1-holes. Persistent homology provides a framework for uncovering complex clinical patterns, offering an alternative to understanding resistance mobility using presence-absence data, which could be obtained through methods beyond genomic sequencing.

## 1 Introduction

Antimicrobial Resistance (AMR) is a critical global health threat, comparable in severity to the challenges posed by HIV and malaria (Antimicrobial-Resistance-Collaborators, 2022). The collection of gene families conferring AMR, known as the resistome, is especially important for studying clinically relevant bacteria. The Critical Assessment and Massive Data Analysis (CAMDA) microbiological challenges have historically focused on understanding the origins of microbial samples using their taxonomical composition (Walker et al., 2018; Zhang et al., 2021). In 2023, the challenge promoted identifying the city of origin of hospital-associated bacteria by analyzing their resistomes and microbiomes from nearby cities' subways. Transport microbiome data may offer a valuable proxy for studying urban microbial life, including hospital bacteria. Previous studies have considered AMR patterns to distinguish between city patterns in urban microbiomes (Casimiro-Soriguer et al., 2019; Zhelyazkova et al., 2021; Danko et al., 2021). However, evolutionary aspects, such as evidence of deviations from hierarchical datasets, remain underexplored. Our study aims to explore the topological features of the resistome to provide insights into non-hierarchical processes, such as Horizontal Gene Transfer (HGT) in nosocomial settings, including CAMDA 2023 datasets; see Section 4 for details on the datasets.

Genes in the resistome are often encoded inside bacterial genomes in mobile genetic elements (MGE). Horizontal gene transfer (HGT) occurs when one bacterium transfers DNA to another, which does not result from cellular division, and the transfer occurs through a process different from traditional vertical inheritance. Hospital studies have shown that antibiotic resistance elements can be transferred even among bacteria of different genera (Evans et al., 2020). Topological data analysis (TDA), particularly persistent homology, can characterize HGT in pathogenic bacteria (Emmett and Rabadán, 2014; Rabadán and Blumberg, 2019). When analyzed with persistent homology, hierarchical data show no holes in their structure (Chan et al., 2013). Populations composed of descendants of one ancestor through mutation, reproduction, and natural selection cycles are hierarchical data and can be represented as a dendrogram and visualized as a tree. Non-vertical inheritance events such as HGT perturb hierarchical data, allowing persistent holes. Remarkably, as we will show, not all spaces without holes are tree-like.

The CAMDA dataset comprises a presence-absence table of 505 AMR markers detected across 146 bacteria, including *Enterobacter hormaechei* (8), *Escherichia coli* (14), and *Klebsiella pneumoniae* (124). These bacteria were isolated from the same hospital over several years. The precise location of the hospital in the USA was not disclosed before the end of the Challenge. Additionally, urban metagenomes were provided by the MetaSUB consortium (Mason et al., 2016). Before applying TDA to CAMDA resistomes, we followed two strategies to understand events where AMR was horizontally transferred among bacteria. First, we simulated HGT on AMR elements in hierarchical data to describe the effect of length in HGT and the number of mobile elements on persistent homology analyses. Second, we described topological holes in cases of HGT between the resistomes of two hospital-associated bacteria, including *E. coli* and *K. pneumoniae* (Evans et al., 2020).

In mathematics, especially in topology and TDA, "holes" refer to empty spaces within a shape or structure. These holes are areas enclosed by the shape but not filled. A simple example of a 1-hole is a circle, where the space is enclosed by the circumference but not filled in. When examining a dataset that represents points on a plane, we may first notice small clusters that form temporary holes. As we zoom out or change the scale, only the more significant, persistent 1-holes remain visible, indicating stable features in the data across different scales. Persistent holes aid in identifying meaningful structures in data as they reveal which features are robust across varying levels of observation. Introductory reviews on mathematical foundations provide deeper insights for interested readers (Chazal and Michel, 2021; Bukkuri et al., 2021). TDA proves valuable in biomedicine applications by identifying complex interactions between diseases and metabolic features (Platt et al., 2024), analyzing signals to detect abnormalities such as arrhythmias (Dindin et al., 2020), discovering intrinsic structures in neural activity (Giusti et al., 2015), and identifying and classifying tumors and diseases in oncology (Bukkuri et al., 2021). Additional applications include analyzing complex networks (Horak et al., 2009), identifying recombinations in hundreds of human genomes (Cámara et al., 2016), and detecting patterns or structures in 3D shapes (Carrière and Rabadán, 2020; Singh et al., 2007).

Finally, we applied TDA to characterize the resistomes of the CAMDA hospital samples of *E. hormachei, E. coli*, and *K. pneumoniae*. In a world where the threat of epidemics looms and the indiscriminate use of antibiotics continues to rise, our work demonstrates that HGT can be inferred from the resistome's presence/absence features without needing the exact DNA sequence. This approach highlights the potential of TDA in uncovering complex genetic interactions and evolutionary events. It aligns with recent collaborative efforts that showcase the utility of TDA in pangenomic and genomic studies (Contreras-Peruyero et al., 2024).

## 2 Methodology

### 2.1 Simplices, holes, and connected components

Persistent homology is a fundamental tool in topological data analysis (TDA), a field rooted in algebraic topology. Currently, TDA is used to analyze the geometric and topological properties of data. This approach examines simplicial complexes, which are topological structures formed by the union of vertices, edges, triangles, tetrahedra, and their combinations. The elements of a simplicial complex are known as simplices and are geometrically represented as follows: vertices are 0-dimensional simplices (0-simplices), edges are 1-dimensional simplices (1-simplices), and triangles are 2-simplices, among others. The features studied in simplicial complexes include connected components and holes. Connected components are groups of dots connected by a path of edges, while holes exist in various dimensions. For example, a 1-hole (or 1-dimensional cycle) is a closed collection of edges (1-simplices) that form a loop without a filling inside. A circumference is an example of a 1-hole since it only comprises the perimeter of the circle (i.e., not the inside). A 2-hole is a 2-dimensional cavity formed by triangles (2-simplices). An empty tetrahedron, which definitionally has triangles as faces, is an example of a 2-hole.

The number of connected components and holes of different dimensions are descriptive features of the simplicial complex. Using homology, we can compute the persistent homology groups for a simplicial complex (see Supplementary material Section 1 or (Dey and Wang, 2022) Section 3.2 for a formal definition); the dimensions of these groups are called the Betti numbers. The interpretation of these Betti numbers is as follows: The Betti number β_0_ represents the number of connected components in the simplicial complex, β_1_ represents the number of 1-holes, and β_2_ represents the number of 2-holes, similarly for higher dimensions.

### 2.2 Persistent holes in a filtered simplicial complex

**Figure 1:**
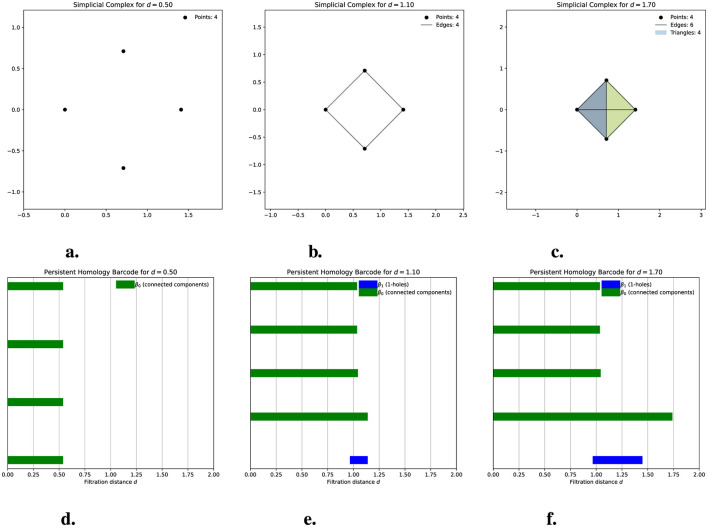
The evolution of a simplicial Vietoris-Rips complex for four points in the plane is shown. **(a–c)** illustrate the graphical representation of the simplicial complexes at distances *d* = 0.5, *d* = 1.1, and *d* = 1.7, respectively. Note that the circles centered on the points have a radius of *d*/2. **(d–f)** display the corresponding persistence barcodes. For *d* = 0.5 **(a)**, we observe four connected components, each representing one of the isolated points. This is reflected in the barcode **(d)**, where four bars correspond to these four connected components, each alive at *d* = 0.5. At *d* = 1.1, the simplicial complex **(b)** consists of four vertices and three edges, forming a 1-hole in the complex. In the barcode **(e)**, three of the bars corresponding to connected components have ended, leaving one persistent bar that indicates a single connected component at this distance. A new blue bar also appears, representing the birth of the 1-hole, which remains open at this stage. For *d* = 1.7, the simplicial complex **(c)** includes four triangles (though only two are visible) that fill the previously existing 1-hole. In the barcode **(f)**, a single bar for the connected component remains alive, while the blue bar representing the 1-hole has ended at the moment the triangles formed and filled the hole.

Now that we have described the structure and characteristics of simplicial complexes, we can explore their construction. In this article, we work with the Vietoris-Rips complex, restricted to dimension 2. Suppose we have a finite set of points in a metric space and a value *d*≥0. These points will represent the vertices of our simplicial complex, i.e., the 0-simplices. The edges, or 1-simplices, between each pair of points are formed when the distance between them is less than or equal to *d*; that is, if we consider circles of radius *d*/2 centered at the points, we have 1-simplices if the circles intersect. The 2-simplices, or triangles, are formed when, for every triplet of points, there is an edge between them. In this way, we have a simplicial complex for each value of *d*≥0. The collection of all these simplicial complexes is called a filtered simplicial complex. This allows us to observe how the simplicial complex evolves as we adjust the filtration parameter (as the value *d* increases). For each value of *d*, we compute the Betti numbers and present them in a barcode called a persistence barcode. In a barcode, we can observe how the simplicial complex evolves, which provides information about changes in the connected components and holes. In all cases, the barcode presented is the result when the maximum distance between all points is reached, except in Figure 1, where we show intermediate steps of the simplicial complex and its barcode up to that point. In a barcode, the left endpoint of the bars is referred to as a birth, and the right endpoint is referred to as the death. We usually refer to a particular topological feature (connected component or 1-hole) being born and dying at those points. The length of a bar is known as its persistence. Thus, on the x-axis, we observe the variation of the filtration parameter. At the same time, the y-axis does not possess a scale because it only stacks bars representing connected components and holes that demonstrate their birth and death. Typically, in barcodes, the Betti numbers of different dimensions are represented by different colors; for example, in Figure 1, green bars are related to β_0_ (connected components), and blue bars are related to β_1_ (1-holes). If another color appears, it represents holes of higher dimensions.

Figure 1 will help us understand these previous concepts. In Figure 1a, we see that the simplicial complex for *d* = 0.5 (i.e., the radius of the circles is *d*/2 = 0.25) consists of only four vertices because the distance between them exceeds 0.5. Consequently, the balls do not intersect, and in its persistence barcode, we observe four bars representing the connected components that persist from distance 0 to distance 0.5. In Figure 1b, where the distance is 1.1 (i.e., the circle's radius is once again half of this distance), the simplicial complex includes four vertices and four edges. The barcode indicates that three bars have already died, leaving only one for this distance, which signifies that we now have just one connected component. We also observe a blue bar representing the 1-hole formed in the simplicial complex, which was born at *d* = 1 and remains alive at *d* = 1.1. Finally, for *d* = 1.7, the simplicial complex now comprises four vertices, six edges, and four triangles (though only two triangles are visible in the diagram). The barcode shows that the 1-hole has died, meaning the triangles have filled it in.

### 2.3 Persistence barcodes on hierarchical data

Hierarchical data are relevant because it is known that their persistence barcode contains no holes (Rabadán and Blumberg, 2019). Here, hierarchical data were simulated with populations of binary chains to study their topological properties. To emulate CAMDA resistomes of bacteria collected at Baltimore Hospital, the size of the simulated binary chains was set to 505 positions, where the first chain contained 180 ones and 325 zeros. Beginning with this chain, we simulated hierarchical data using our custom-built Python script, simulator resistome, accessible in our GitHub repository.

A hierarchical population of computationally simulated resistomes was generated from this original chain with two descendants per generation. In each reproduction step, a zero can change to one, or a one can change to zero, with a probability of $\frac{1}{505}$. This rate was selected to achieve approximately one change in each generation. This is not a mutation rate, as we are modeling the presence and absence of gene families, not punctual mutations in the DNA. Instead, it can be viewed as simulating gene gain and loss one at a time. We adopt a simplified model where changing a position twice is forbidden; therefore, the gain and loss of the same gene are not allowed. With this process over seven generations, we obtained a population of 128 simulated bacterial resistomes stored in a resistance marker matrix. Each row represents a chain, and each column with a one in the *i*−th position signifies the presence of the *i*−th gene family in that bacterial sample. The simulation required Python libraries, including NumPy 1.21.6, Seaborn 0.12.2, Matplotlib 3.5.3, SciPy 1.7.3, GUDHI 3.6.0, and NetworkX 2.2.

Pairwise distances were calculated for each pair of chains. A distance matrix from a resistance marker binary matrix was constructed using the "Hamming" metric, which counts the number of differences by position between two chains. The distance was calculated with pdist from scipy, which returns a normalized distance matrix. Vietoris-Rips complexes were constructed using the Python Gudhi package 3.6.0, with the maximum distance set to 2 and a dimension of at most 3. Betti numbers and persistent barcodes associated with variations in the Hamming distance were calculated for simulated hierarchical data.

### 2.4 Persistence barcodes of nonhierarchical data

To perturb the hierarchical structure of the data, in a subset of *m* computationally simulated resistomes, a set of *k* contiguous resistance markers (columns) is turned to one, regardless of their previous state. This change biologically corresponds to the sudden gain of these *k* gene families in *m* bacteria, consistent with an HGT event. In subsequent simulations, we vary *m*, the number of bacteria that acquire the HGT, and *k*, the number of acquired resistance markers. Finally, to emulate the event in which bacteria from different taxonomic lineages are involved, we allow two different binary chains to be ancestors of two populations. Betti numbers and persistent barcodes associated with Hamming distance were calculated similarly to hierarchical data.

### 2.5 Persistence barcodes of nosocomial data

**Figure 2:**
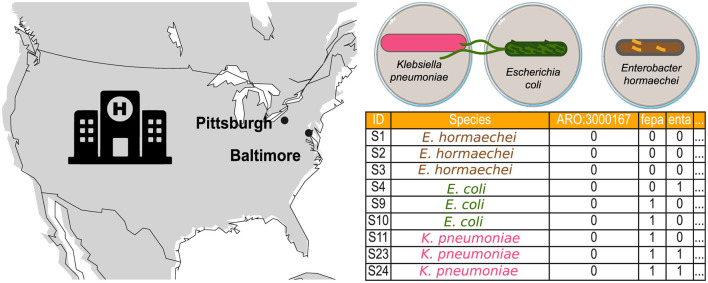
Bacterial data consist of AMR profiles isolated from a hospital in Baltimore and bacterial genomes from a hospital in Pittsburgh. The Baltimore data are provided by the CAMDA challenge, which includes resistance markers for three bacterial species: *K. pneumoniae, E. coli*, and *E. hormaechei*.

This study considers two nosocomial datasets to investigate their topological properties. The first dataset consists solely of presence-absence information about AMR genes of bacteria isolated from a Baltimore hospital and provided by the CAMDA Challenge (Figure 2). The second dataset consists of twelve genomes from bacteria isolated in a Pittsburgh hospital Table 1. In the CAMDA dataset, a binary table marks the presence of resistance genes of bacteria isolated in Baltimore with one and their absence with zero. The 146 resistomes from *E. hormaechei, E. coli*, and *K. pneumoniae* were represented as binary sequences. Pairwise Hamming distances were calculated for these binary sequences, and the resulting distance matrix was then used to generate the persistence barcode.

**Table 1:** Bacterial isolates from hospital patients .

**RefSeq assembly**	**Genomes**	**Strain**
GCF_012952615.1	*E. coli*	EC00609
GCF_012952605.1	*E. coli*	EC00668
GCF_012952555.1	*E. coli*	EC00678
GCF_012952545.1	*E. coli*	EC00690
GCF_012952535.1	*E. coli*	EC00701
GCF_012952515.1	*K. pneumoniae*	KLP00149
GCF_012952505.1	*K. pneumoniae*	KLP00155
GCF_012952415.1	*K. pneumoniae*	KLP00215
GCF_012952405.1	*K. pneumoniae*	KLP00187
GCF_012952385.1	*K. pneumoniae*	KLP00213
GCF_012952365.1	*K. pneumoniae*	KLP00218
GCF_012952465.1	*K. quasipneumoniae*	KLP00177

In the second dataset, we included a known case of HGT between two genera (Evans et al., 2020) in a Pittsburgh hospital (Figure 2). The clinical case documented that gene families in the IncF plasmid were shared between two *E. coli* strains and one *K. pneumoniae* strain. Genomes from the same study representing the genera *Escherichia* and *Klebsiella* were downloaded from NCBI (Table 1). The strains involved in the HGT event are EC00701, EC00678, and KLP00215. Genomes were processed and converted into a binary matrix, with bacterial genomes as rows and gene families as columns. First, FASTA files were uploaded to the Bacterial And Viral Bioinformatics Resource Center (BV-BRC) 3.32.13a platform (Olson et al., 2023) to obtain the binary matrix. Then, the genomes were functionally annotated via the RAST platform using the RASTtk toolkit (Brettin et al., 2015). A gene family table encompassing all the genomes was constructed using the Comparative Systems tool. The table was then sorted based on the presence of gene families in the EC00701 strain. From the entire table with 13165 columns representing gene families, we selected 500 columns containing the 123 gene families of the IncF plasmid involved in the reported horizontal transfer. These binary sequences encode the presence-absence information of gene families across the twelve *Escherichia* and *Klebsiella* genomes from the Pittsburgh hospital. We calculated pairwise Hamming distances using these binary sequences to create the distance matrix. Finally, a persistence barcode was constructed to describe the variation in the number of connected components and loops (1-holes) across the range of possible Hamming distance values.

## 3 Results

To leverage persistent homology from TDA for detecting HGT of antibiotic resistance, we first examined how persistent homology behaves with hierarchical data. Hierarchical data refers to a structure where each related item indicates a parentchild relationship.

### 3.1 Hierarchical data simulations show no 1-holes in persistent homology analyses

The persistence barcodes of the hierarchical data do not contain 1-holes; this directly results from Theorem 5.2.1 in Rabadán and Blumberg (2019). To illustrate, we present a population of binary sequences composed of 0s and 1s obtained through a hierarchical process. In each generation, sequences replicate into two new sequences identical to the original, except for potential changes at one position. This forward-in-time simulation model passes on identical sequences, except for occasional variations introduced at a fixed probability. These variations, once acquired, are inherited by subsequent generations, resulting in a hierarchical process where each element (node) can have multiple child nodes. However, each child node has only one parent node. The simulation began with a single sequence of 505 positions, where 25% of the positions were ones. After seven repetitions, this process produced 128 sequences, considering only the last generation. Importantly, it was assumed that once a position changed, it remained fixed in all subsequent generations. Tree structures are the standard for modeling vertical or clonal evolution. Although this model does not fully capture the complexity of prokaryotic genome evolution, it provides a starting point for exploring the topology of perturbed hierarchical processes. This applies across scales, whether variation stems from point mutations or from the gain and loss of genes.

**Figure 3:**
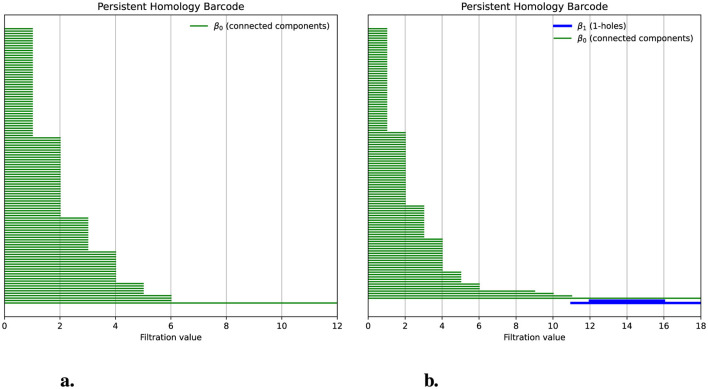
Deviations from a hierarchical process produce 1-holes. **(a)** The persistence barcode indicates no 1-holes for a population of 128 chains simulated hierarchically. Green bars represent the persistence of the connected components; for filtration values greater than six, only one bar remains, indicating that the distance between all chains is below a threshold. **(b)** The persistence barcode, after introducing a deviation from the hierarchical process, shows two 1-holes. Blue bars indicate the birth, death, and persistence of 1-holes.

To obtain the persistence barcode, we counted the differences between each pair of chains using the Hamming distance and then applied persistent homology. The resulting barcode showed the absence of 1-holes; the Betti number β_1_ is 0, as shown in Figure 3. We can also observe that we start with 128 bars, each representing one chain, which indicates that β_0_ = 128 corresponds to 128 connected components for *d* = 0. As the distance increases on the x-axis, the bars merge, indicating that chains sharing a common ancestor are becoming connected. By distance 6, only one connected component remains, where β_0_ = 1. Next, we examine how the 1-holes, represented by the Betti number β_1_, change when there is a deviation from the hierarchical process (see Figure 3). To explore this, we will simulate such a deviation.

### 3.2 Deviations from the hierarchical process reveal the presence of 1-holes in persistent homology analyses

#### 3.2.1 Simulation of deviations from the hierarchical process in one population

To simulate a deviation from a hierarchical process, we selected chains from our population and made them share a common segment. We used the following parameters to construct the population: A group consists of three chains (group_size = 3), and the size of the shared segment is equal to 60 positions (num_positions_to_change = 60). The choice of a group size of three relates to the minimum required to ensure the formation of a 1-hole when constructing a Vietoris-Rips simplicial complex. Similarly, the segment size of 60 positions represents approximately 12% of the total chain length. This value was selected to ensure that the segment is neither too small, risking undetectability of the 1-hole, nor too large, as it could dominate the chain. A random position was selected between 1 and the total chain length minus the segment size as the starting point. We then changed all the positions from this starting point to the starting point plus the segment size to 1. In this way, we simulated that these three chains acquired this section through a non-hierarchical process. These chains form a group whose distances do not satisfy a tree-like structure.

**Figure 4:**
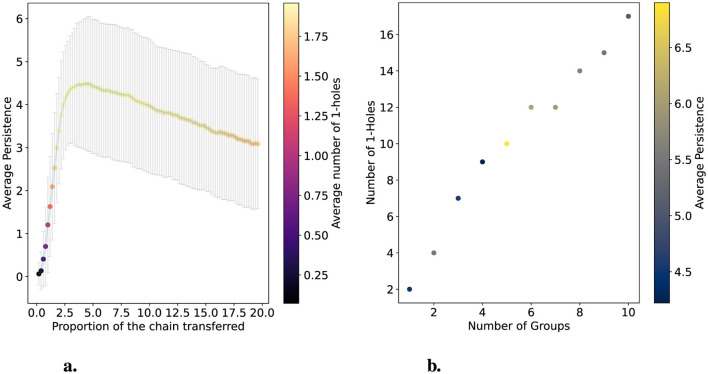
Simulations varying the size of a transferred segment and the number of groups that receive the transfer. **(a)** shows the deviation from hierarchical data in one group of three chains. The x-axis varies the proportion of the transferred segment in terms of the total size of the chain (505 positions). The average persistence of 1-holes is shown on the y-axis. One hundred populations were simulated for each point, and error bars represent variance. Colors with a scale on the right y-axis indicate the average number of 1-holes obtained for each size. **(b)** shows the changes in the number of 1-holes and their average persistence as the number of groups undergoing segment transfer varies. In this case, the number of transferred positions was fixed at 60, and three genomes integrated the groups.

When applying persistent homology to this modified population, we observed the presence of two 1-holes, indicating a Betti number β_1_ = 2. In Figure 3b, we see two blue bars with persistence values of 4 and 7, which represent the persistence of these 1-holes. Our simulations demonstrate that when a population following a hierarchical process exhibits deviations from that process, these deviations can be detected through the presence of 1-holes when applying persistent homology. In this case, the persistence has an average value of 5.5 per bar. However, to determine whether this average is associated with the size of the shared segment, we conducted simulations where the transfer size varied from 0 to 20% of the total chain length. Additionally, for each of these sizes, we performed 100 simulations with different random seeds. The results are displayed in Figure 4a.

In Figure 4a, we observe that varying the size of the segment transferred in terms of the percentage of the chain full size (505 positions) causes the average persistence of 1-holes to grow rapidly until it reaches its maximum, after which it decreases slowly. On average, the maximum persistence is around 4.5 when the transfer size represents 4% to 5% of the chain size. Furthermore, the figure shows that when the transfer size represents less than 3% of the chain size, the average number of 1-holes is 0 or 1. Outside of this range, the average number of 1-holes is 1.75. Specifically, for each size, 85 out of 100 simulations have two 1-holes. This is because the maximum number of 1-holes is 2 and the minimum is 0. In Figure 4b, we simulate another hierarchical population with 128 chains, setting the deviation size in the hierarchical process to 60 and fixing the group size at three while varying the number of groups. In this simulation, we observe that there are almost always two 1-holes for each group, except when there are six or seven groups, where twelve 1-holes are detected. This suggests that within a population, the transfer of a segment among a group of 3 chains consistently produces two 1-holes.

Processes leading to non-tree-like structures in populations, such as those observed in our simulations, can include transformation, transduction, and conjugation in bacteria, reassortment, and homologous recombination in viruses (Rabadán and Blumberg, 2019). These deviations from hierarchical structures contribute to the emergence of 1-holes, as captured through persistent homology. We will now present simulations with a population consisting of two different groups and examine whether the transfer of segments between groups can again be detected using persistent homology.

#### 3.2.2 Simulations of deviation from the hierarchical process between two populations

**Figure 5:**
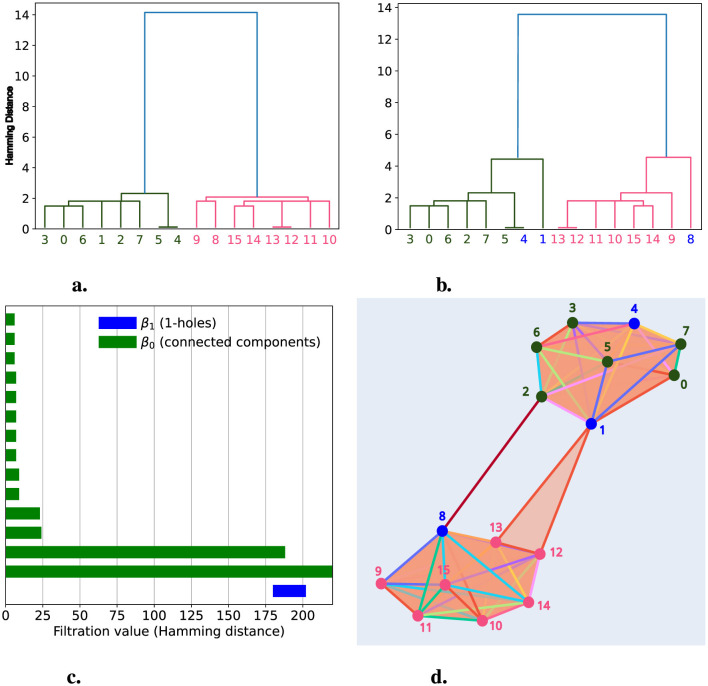
Deviations from Hierarchical Processes with Two Populations. We simulated two hierarchical populations, each consisting of eight chains represented as vertices colored pink and green. In **(a)**, we present a dendrogram with the 16 chains, where the x-axis shows the labels for each chain, and the colors indicate the population to which they belong. In **(b)**, we display a dendrogram after simulating a deviation from the hierarchical process involving chains 1, 4, and 8 (shown in blue). **(c)** presents the persistence barcode after simulating the deviation from the hierarchical process. Here, the green bars represent the persistence of connected components, while the blue bars represent the persistence of 1-holes. In **(d)**, we show the simplicial complex representation at filtration level *d* = 184, where we can observe the formation of a 1-hole created by chains 1, 2, 8, and 13.

We simulated two hierarchical populations of chains, each satisfying a tree structure, composed of eight chains per population across three generations. In Figure 5a, we show the dendrogram for this group of 16 chains, where the two populations are clearly separated. We then selected chains 4 and 1 from population 2 (green) and chain 8 from population 1 (pink), transferring a section among these three chains, as described in the previous section. The resulting dendrogram is shown in Figure 5b, where we observe a change in the tree structure, with the changes linked to the chains (blue) involved in the segment transfer.

After applying persistent homology, we detected a 1-hole with a persistence of 14, which was born at distance 184 and died at distance 198, as shown in Figure 5c. This persistence barcode shows two bars representing the connected components (β_0_) with the highest persistence: 184 and infinity. These bars correspond to each population, which groups separately until distance 184, where they merge just as the 1-hole, formed by chains 1, 2, 8, and 13, is born, as illustrated in Figure 5d. This figure provides a graphical representation of the simplicial complex at *d* = 184, where the two populations are shown as distinct groups connected by the 1-hole. Once again, TDA through persistent homology enables us to detect segment transfers between chains, this time between different populations.

Following the analogy established in previous sections, we could hypothesize that we have two bacterial populations—one of *Escherichia* and one of *Klebsiella*—where HGT occurs between the populations. Persistent homology can be used to detect this horizontal transfer through the presence of 1-holes, as we will demonstrate with data from hospital isolates in the following section.

### 3.3 AMR acquired by HGT in nosocomial data show persistent 1-holes

#### 3.3.1 Inter-species antibiotic resistance HGT in Pittsburgh hospital shows persistent 1-holes

**Figure 6:**
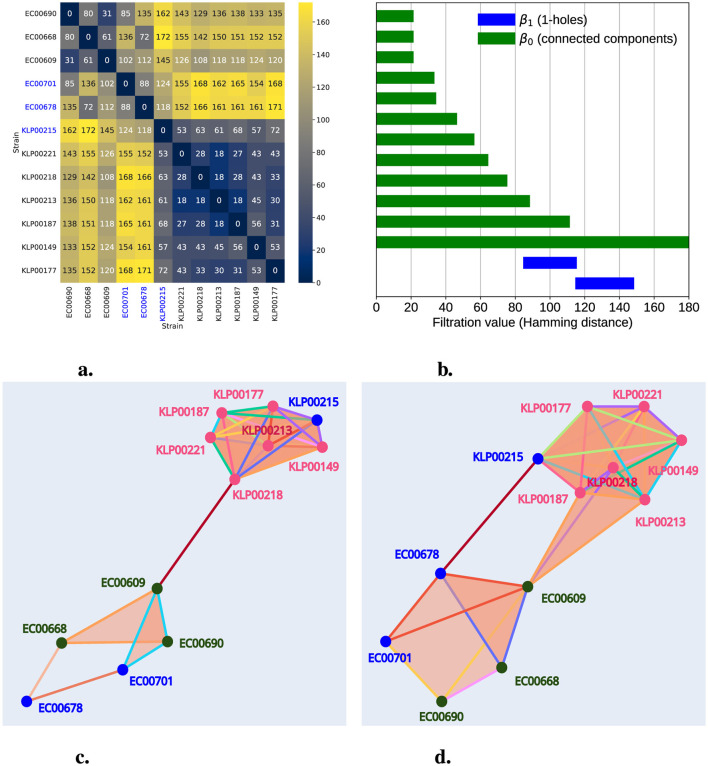
Persistent homology captures HGT in AMR Nosocomial Data. In **(a)**, we present a heatmap of a distance matrix calculated from a gene count table, including the IncF plasmid, for the strains listed in Table 1. The numbers and color gradient represent the distances, with blue-highlighted labels indicating strains exhibiting HGT. **(b)** shows the persistence barcode, where green bars represent the persistence of connected components and blue bars indicate the persistence of 1-holes. **(c, d)** illustrate representations of the filtered simplicial complex at filtration levels 108 and 119, respectively. Pink labels represent *Klebsiella* strains, green labels indicate *Escherichia* strains, and blue labels highlight strains involved in HGT. In **(c)**, we observe a 1-hole formed exclusively by *Escherichia* strains. In **(d)**, we observe another 1-hole involving both *Klebsiella* and *Escherichia* strains.

Hospitals are urban sites of interest in microbial surveillance. They aim to identify HGT and antibiotic resistance, especially in clinically relevant bacteria. Genes conferring antibiotic resistance can be shared by HGT, even among bacteria of different genera (Evans et al., 2020; Constantinides et al., 2020). In 2020, Evans et al. (2020) scanned 2,173 bacterial genomes isolated between 2016 and 2018 from the University of Pittsburgh Medical Center in one hospital. At least ten distinct mobile elements were tracked across the genomes, with identical DNA regions shared between bacteria of different genera, suggesting evidence of HGT. One particular clinical case illustrates how one *E. coli* ST69 isolate (EC00678) and one *K. pneumoniae* isolate (ST405) were collected from Patient A, sharing the same mobile genetic element (MGE). Patient B also contains an almost identical mobile element in a *E. coli* isolate (EC00701). Patients A and B were in contiguous rooms, though not simultaneously. The shared MGE is the IncF plasmid, which contains approximately 113.6 Kb. IncF is an *Enterobacter* plasmid with resistance genes often found in *E. coli* (Rozwandowicz et al., 2018). Nevertheless, in this clinical case, IncF was found in *E. coli* and *K. pneumoniae*, two bacteria of different genera. In Evans's study, genomes of particular interest were sequenced again with long reads, improving the understanding of how AMR gene families are distributed in mobile elements (Table 1). From these high-quality genomes, we downloaded all sequences from genera *Klebsiella* and *Escherichia* from NCBI (Table 1). To analyze their persistent homology, we selected 500 gene families, including those in the IncF plasmid found in *E. coli* strain EC00768. Figure 6a illustrates this, divided into four regions. The redder regions indicate the distances between different species of *Escherichia* and *Klebsiella*. Notably, the fourth quadrant represents the distances among *Klebsiella* strains, while the first quadrant is for *Escherichia*. In this latter quadrant, we can observe some reddish hues related to the distance between the strains where the horizontal transfer occurred.

Utilizing Hamming distances presented as a heatmap in Figure 6a, we constructed a Rips simplicial complex and calculated persistent homology with a maximum dimension of 3; we identified the presence of two 1-holes. These appeared at filtration levels 88 and 118 and dissipated at 112 and 145, respectively, as shown in Figure 6b. Additionally, this plot reveals an infinite persistence bar in β_0_ (connected component) and another with a persistence of 108. As expected, this latter observation is linked to our data being clustered into two groups: *Klebsiella* and *Escherichia*. Furthermore, Figure 6c provides a graphical representation of the simplicial complex filtered at filtration level 108, showing the presence of the first 1-hole (which emerged at 88), formed by EC00668, EC0678, EC00701, and EC00690. At this stage (filtration level 108), a connected component dissipated, and an edge connecting KLP00221 and EC00609 emerged, bridging the groups formed by *Klebsiella* and *Escherichia*. Finally, Figure 6d illustrates the simplicial complex at filtration level 119, where we can observe the second 1-hole (emerging at 119) formed by EC00678, EC0609, KP00215, and KP00187.

Thus, TDA enabled us to detect the presence of 1-holes representing horizontal transfer among EC00701, EC0678, and KP00215, a phenomenon previously confirmed by the high sequence identity of the IncF plasmid family region.

#### 3.3.2 The resistome of bacteria isolated from a Baltimore hospital has 1-holes

Every year, the CAMDA community of interest provides an open big data challenge. The CAMDA 2023 Anti-Microbial Resistance Prediction Challenge comprises two data sets to study AMR patterns. First, CAMDA supplied a presence/absence table of 505 AMR markers from 146 bacteria isolated and sequenced from a single hospital over several years; see Figure 2. This hospital's resistome data contains AMR markers from 14 *E. coli*, 124 *K. pneumoniae*, and 8 *E. hormaechei*. Second, shotgun metagenomes from 2016 and 2017 collected from sixteen cities worldwide were provided by the MetaSub consortium (Mason et al., 2016). The hospital belongs to one of the cities in the United States, but the exact location was not disclosed before the end of the challenge. We attempted two approaches to obtain information from CAMDA data: (i) identifying the topology of the hospital resistome table, and (ii) searching for differential genes between cities using species-level pangenomes developed with public genomes from dates and cities following CAMDA data (available in the Supplementary material).

**Figure 7:**
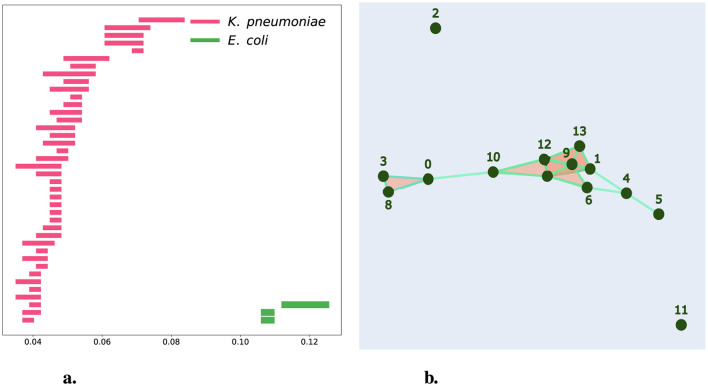
Persistent Homology for CAMDA Bacterial Resistance Marker Data. **(a)** shows a barcode representation for samples from hospitals. Pink bars represent *K. pneumoniae*, while green bars represent *E. coli*. **(b)** provides a geometrical representation of the simplicial complex at the filtration level of 0.113 for *E. coli* samples from hospitals.

In this study, we aim to identify whether the AMR markers in this hospital resistome table correspond primarily to a hierarchical process. We applied persistent homology to the binary chains that encode the AMR markers of *K. pneumoniae, E. hormaechei*, and *E. coli*, measuring the similarity of the resistomes using the Hamming distance. Unlike previous persistence barcodes, which include the persistence of connected components, in Figure 7a, we present a persistence barcode that exclusively shows the persistence of 1-holes. There are 40 pink bars representing the persistence of 1-holes for the dataset of 124 *K. pneumoniae* strains and three green bars representing the persistence of 1-holes for the 14 *E. coli* strains. We used normalized Hamming distance to enable a comparison between both datasets. Notably, we observe a significantly higher number of 1-holes in the *K. pneumoniae* dataset compared to the *E. coli* dataset, which may be partly due to the difference in sample sizes. We also see variation in the lengths of the persistence of these bars that could reflect the extent of HGT, as suggested by the presence of 1-holes in persistent homology. The lack of holes in *E. hormaechei* could be due to the absence of HGT or the limited data available for *E. hormaechei* for comparison. In Figure 7b, we provide a geometric representation of the simplicial complex at filtration level 0.113, where we observe a 1-hole formed by chains 1, 4, 6, and 9. These chains are potential candidates for HGT. Additionally, we note that at this filtration level, 12 out of the 14 strains are already connected within a single connected component, leaving only strains 2 and 11 unconnected. This suggests that strains 2 and 11 are more distinct from the others at the level of AMR genes.

Once the CAMDA Challenge finished, Baltimore was revealed as the city where the hospital was located. After the persistent homology analysis, our second goal was to understand if, when comparing the resistome hospital table against AMR markers in the metagenomes, there was enough evidence to identify Baltimore as the city of origin of the samples. We constructed eighteen binary tables from the metagenomes, representing the samples of the three bacterial genera in each of the six cities. The presence of AMR markers in the labeled samples is too sparse to accurately determine the mysterious city. We then searched for differential genes between cities using pangenomes elaborated with public genomes from dates and cities similar to the CAMDA samples–; however, no conclusive evidence allowed us to identify Baltimore as the mysterious city.

### 3.4 There are spaces without holes that are not tree-like

In the mathematical aspect of this study, after analyzing the topology of both simulated and actual biological data, we aim to explore the necessary and sufficient conditions that distinguish a tree-like structure from spaces with 1-holes. This section will focus on the interplay between topological features and the underlying data structure, examining how the presence or absence of 1-holes impacts our ability to classify spaces as tree-like. In Section 3.1, we analyzed the case of a hierarchical population represented as a tree, which, in terms of TDA, is indicated by the absence of 1-holes in the persistent barcode. However, some spaces lack a tree structure and also do not possess 1-holes. This section presents an example of such a case and clarifies why the absence of 1-holes should not be interpreted as indicative of a hierarchical population or tree structure.

It is known that in an additive metric space (tree-like) *M*, the *p*-level persistence barcode of the Vietoris-Rips complex is empty for all *p*>0 (Chan et al., 2013), as detailed in Supplementary material Section 2. While being tree-like is sufficient for having Betti numbers equal to zero in higher dimensions, it is unnecessary. Here, we present an example where β_*i*_ = 0 for *i*≥1, yet the space is not tree-like.

Four points satisfy the four-point condition if and only if:


(1) $$\begin{array}{l} d\left(x,\;y\right)+d\left(z,\;t\right)\leq \max\left\{d\left(x,\;z\right)\;+\;d\left(y,\;t\right),d\left(x,\;t\right)\;+\;d\left(y,\;z\right)\right\} \end{array}$$

In 1974, Buneman proved that a graph is a tree if and only if it is connected, contains no triangles, and has a graphical distance satisfying the four-point condition, (Buneman, 1974).

**Figure 8:**
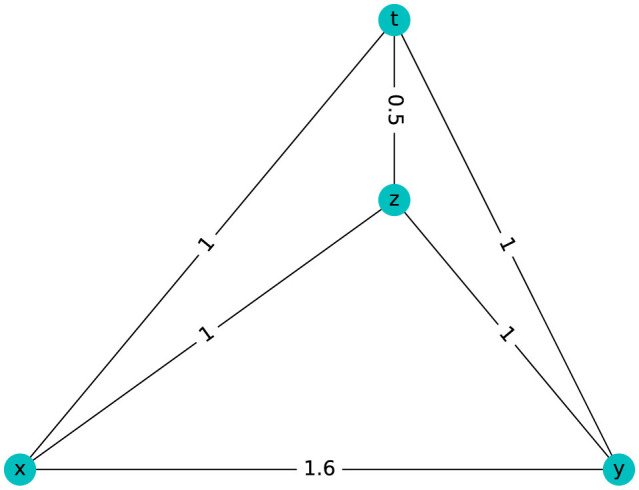
Metrical space with β_*i*_ = 0 for *i*≥1 that is not tree-like.

The graph in Figure 8 has β_*i*_ = 0 but it does not satisfy the four-point condition because:


(2) $$\begin{array}{l} d(x,\;y)\;+\;d(z,\;t)=1.6+.5=2.1>2=\\ \max\{d(x,\;z)\;+\;d(y,\;t)=1\;+\;1=2,d(x,\;t)\\ +d(y,\;z)=1+1=2\} \end{array}$$

Then, Figure 8 is not tree-like.

## 4 Discussion

The original challenge presented by the CAMDA23 community was determining the origin of hospital samples of *K. pneumoniae, E. hormaechei* and *E. coli* by analyzing their resistomes, which consist of around 500 genes, and metagenomes from nearby cities. Despite calculating the AMR profiles in the microbiomes, the low quantity of detectable AMR genes—likely due to the shallow depth of some samples—hindered our ability to characterize the cities based on resistome differences. Therefore, we followed the next challenge: extracting the most information possible from these data. We utilize the topology of the resistomes to investigate evidence of HGT.

To accomplish this, we conducted three stages: a simulation of horizontal transfer by perturbing hierarchical resistomes, a topological description of a known example of inter-generic HGT in a hospital setting, and finally, the identification of topological persistence in the CAMDA data. We aim to understand how many holes form when HGT occurs, how long these holes persist, and which population elements are involved in their formation.

Our results indicate that the persistence of 1-holes varies with the number of genes transferred. Small transfer sizes often result in minimal persistence, suggesting that such events might closely resemble vertical inheritance or be indistinguishable from it due to the similarity of the genomes involved. This underscores the nuanced role of transfer size, alongside other factors like mutation rate and the number of genomes in a group, in influencing the detectability of HGT through TDA. Our findings suggest that HGT can be effectively captured in larger genomic regions, where the transferred genes significantly impact the organism's genomic structure, producing detectable topological features. This aspect highlights the importance of analyzing gene transfers across various scales to understand their evolutionary implications.

In our study, we explore the dynamics of HGT and its detectability through TDA, primarily using persistent homology. Hierarchical data represented as trees models vertical inheritance in evolutionary biology. The core of our analysis relies on the model that tree-like data structures exhibit Betti numbers (β_*i*_) equal to zero for dimensions greater than or equal to one (Chan et al., 2013). This mathematical framework allows us to associate the presence or absence of holes in the data with a deviation from vertical inheritance (Rabadán and Blumberg, 2019), such as HGT in bacterial resistomes (Emmett and Rabadán, 2014). The relationship between persistent homology and data topology in population genomics is threefold: (1) Populations without HGT are hierarchical data and represent tree-like spaces; consequently, they have Betti numbers equal to zero in every higher dimension (i.e., β_*i*_ = 0 for *i*≥1) (Chan et al., 2013). (2) However, we demonstrate that not all populations with β_*i*_ = 0 for *i*≥1 lack HGT, as having Betti numbers equal to zero does not necessarily imply a tree structure. (3) Conversely, the presence of holes (non-zero β_*i*_ numbers for some *i*≥1) indicates a non-tree-like structure, which we model here as evidence of HGT.

**Non-HGT implies a tree-like space with no holes in data**. Evolutionary data modeled in our simulator without gene gain associated with HGT exhibit a tree-like structure devoid of holes. This baseline contrasts with HGT scenarios, further complicating the interpretation of evolutionary dynamics. **A dataset can contain no holes but not have a tree-like structure**. The condition β_*i*_ = 0 for *i*≥1 is necessary but insufficient to assert that data has a tree-like structure. Here, we show an example with no holes (zero Betti number in every dimension) without a tree-like structure, emphasizing the complexity of inferring evolutionary relationships from topological characteristics alone. Also, **HGT does not imply the presence of holes**. In our simulations, not all instances of HGT result in detectable holes in the data structure. This observation aligns with the biological understanding that gene transfer can occur without drastically altering the hierarchical structure of genomes, especially when transfers happen between closely related species or involve a very small number of genes that do not significantly impact the organism's overall genomic architecture. Finally, **HGT is implied by the presence of holes**, since holes mean that the topological structure of the data is not tree-like. These topological features capture gene transfer events that introduce discontinuities or “gaps” in the otherwise smooth narrative of vertical inheritance.

Our research highlights the complexity of detecting and interpreting HGT through topological methods. The interplay between transfer size, genomic similarity, and the methodological nuances of TDA presents a multifaceted challenge in understanding the role of gene transfer in evolution. By meticulously analyzing the persistence of topological features across various scenarios, we contribute to a deeper understanding of the evolutionary processes shaping microbial genomes, providing insights into the subtleties of HGT and its implications for phylogenetic and evolutionary studies.

A potential avenue for future research involves leveraging advances in persistent homology. Some authors have reviewed and described methods to calculate distances between barcodes (Chazal and Michel, 2021) and demonstrated the utility of combining persistent homology-extracted features with machine learning methods to distinguish between datasets (Bukkuri et al., 2021). Applying similar approaches to calculate distances between presence-absence-derived barcodes could enable clustering of datasets with similar horizontal transfer scales, further refining our understanding of HGT dynamics.

In Section 6 of the Supplementary material, we include the **Horizontal Gene Transfer (HGT) Analysis** of the genomes listed in Table 1. First, we conducted an analysis of species and gene phylogenetic trees based on amino acid sequences, which revealed several horizontal gene transfer events, as expected. Subsequently, we performed the same analysis using gene count tables. However, in this case, we did not detect any horizontal gene transfer events between the *Escherichia* and *Klebsiella* groups. Additionally, in Section 3.3, we applied persistent homology to detect the presence of HGT in the same dataset, using the gene count table as input. This approach highlights the advantages of leveraging Topological Data Analysis (TDA) and persistent homology for detecting horizontal gene transfer events.

## Data Availability

The original contributions presented in the study are included in the article/Supplementary material, further inquiries can be directed to the corresponding author.

## Supplementary material

The Supplementary Material for this article can be found online at: https://www.frontiersin.org/articles/10.3389/fmicb.2025.1461293/full#supplementary-material; The scripts are available in the GitHub repository: https://github.com/shadayguerrero/TDA_Horizontal_Genes_Transfer.
