# Supplementary figures and images for: Topological data analysis captures horizontal gene transfer in antimicrobial resistance gene families among clinically relevant bacteria
Source: Front Microbiol. 2025 May 7;16:1461293. doi: 10.3389/fmicb.2025.1461293 (PMC12092391; doi:10.3389/fmicb.2025.1461293)

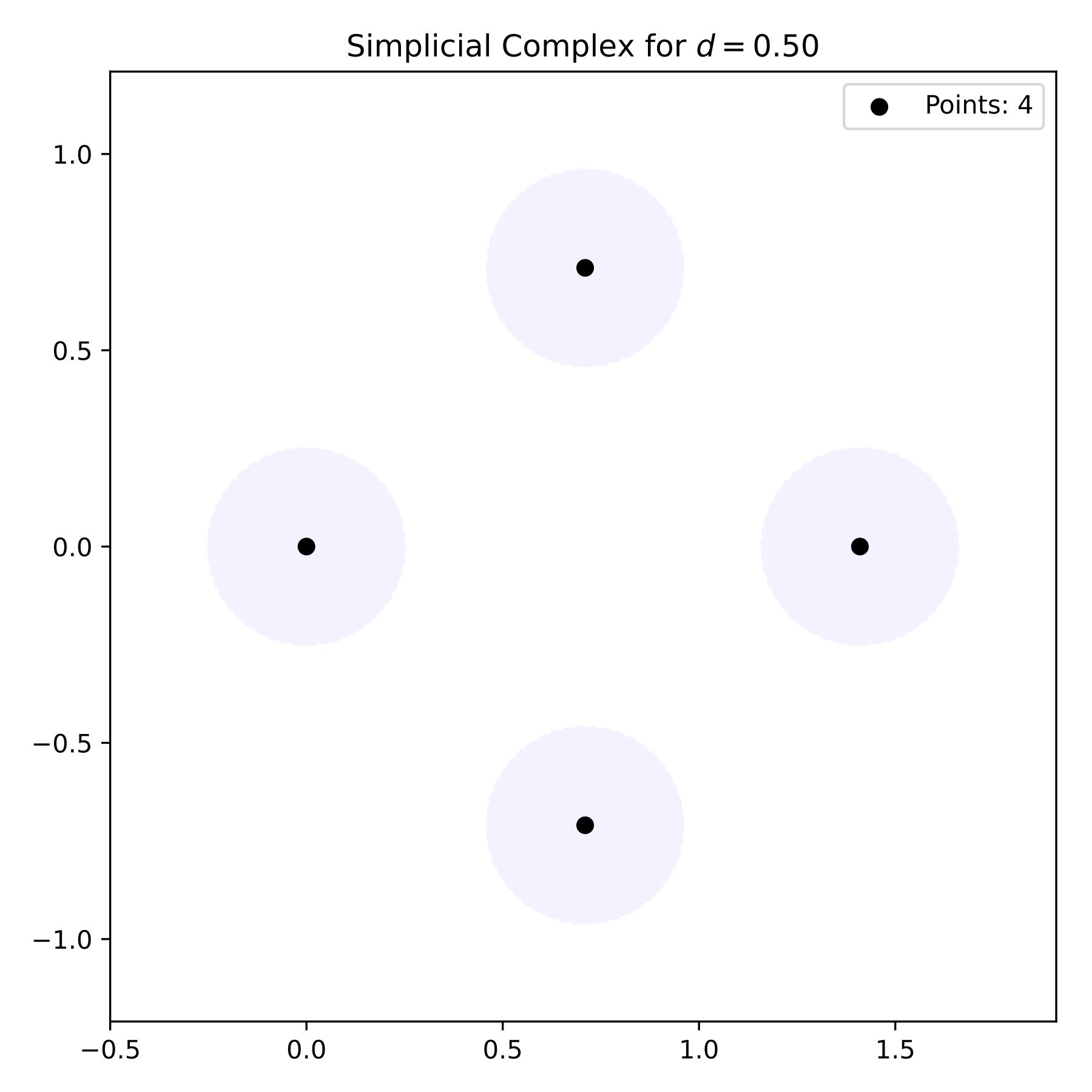

Supplement: Supplementary file 1 [file Data_Sheet_1.zip › TIFF/Figure_1a.tiff]

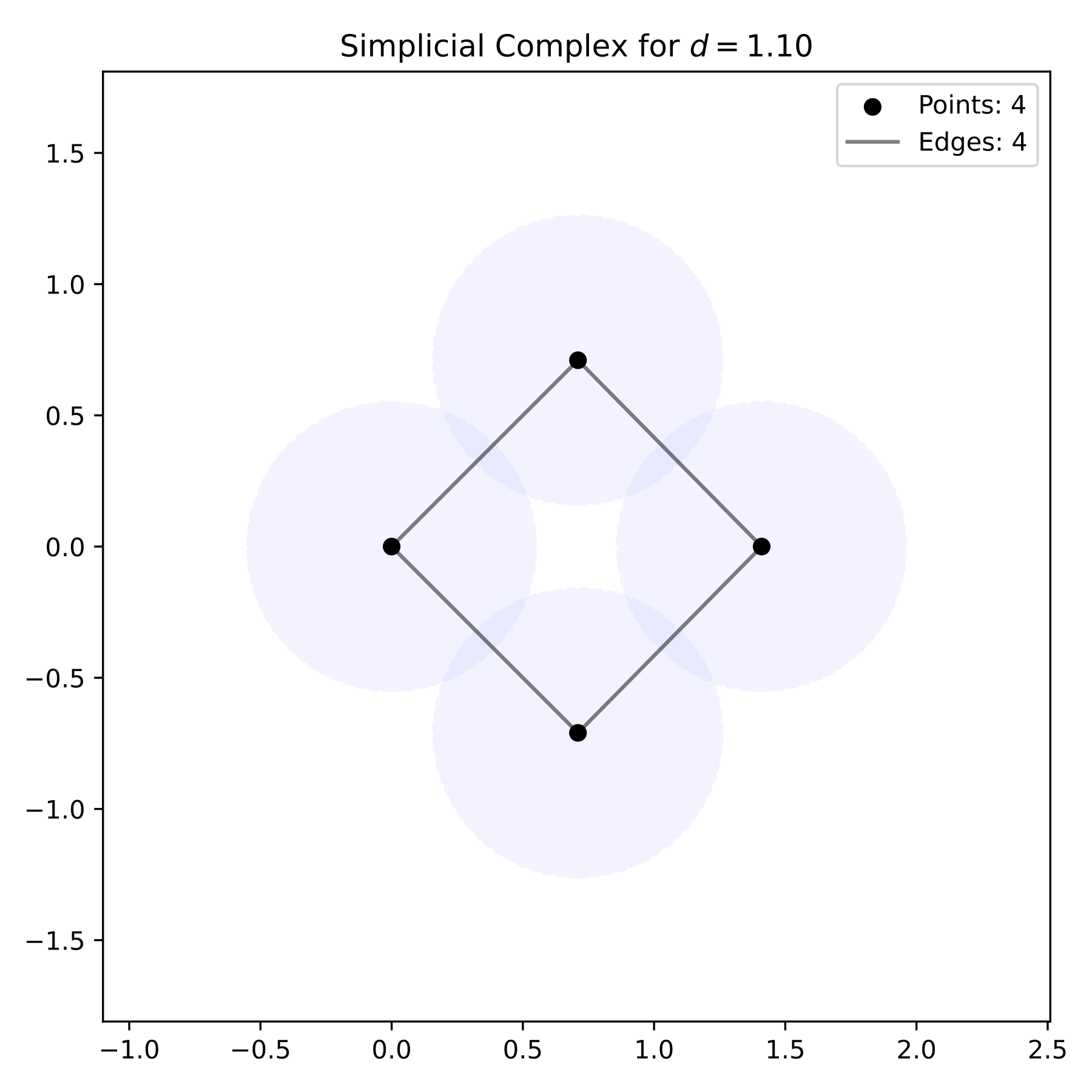

Supplement: Supplementary file 1 [file Data_Sheet_1.zip › TIFF/Figure_1b.tiff]

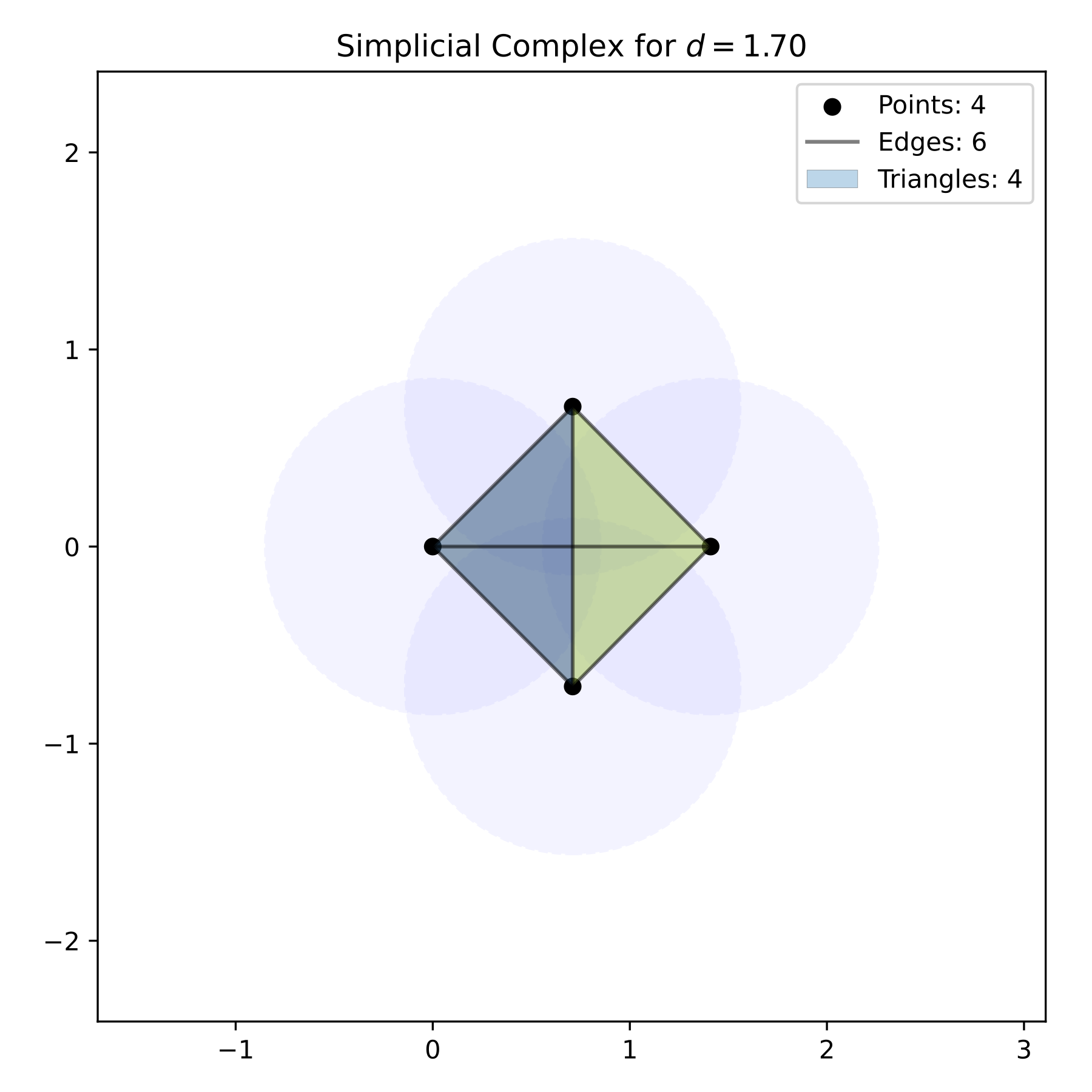

Supplement: Supplementary file 1 [file Data_Sheet_1.zip › TIFF/Figure_1c.tiff]

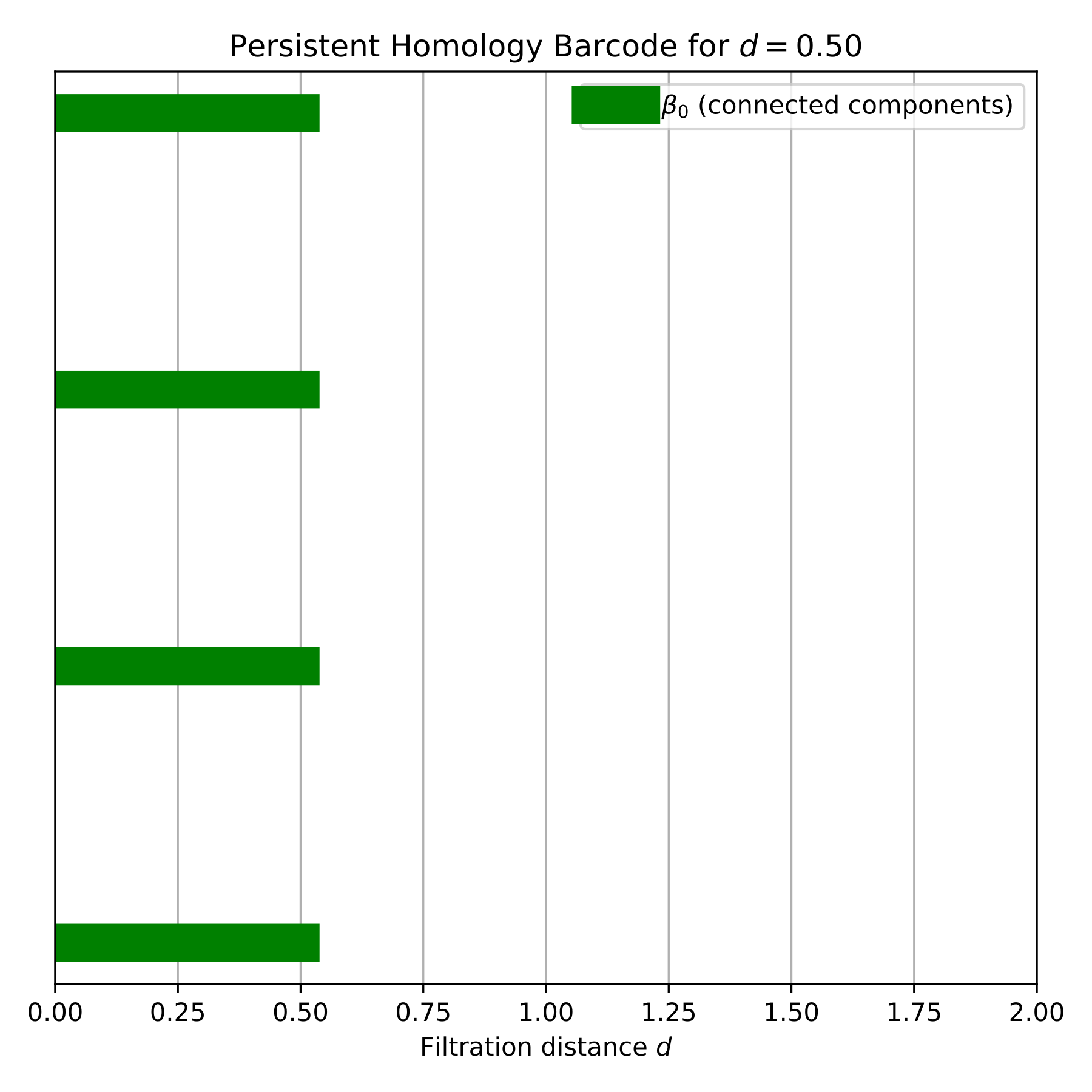

Supplement: Supplementary file 1 [file Data_Sheet_1.zip › TIFF/Figure_1d.tiff]

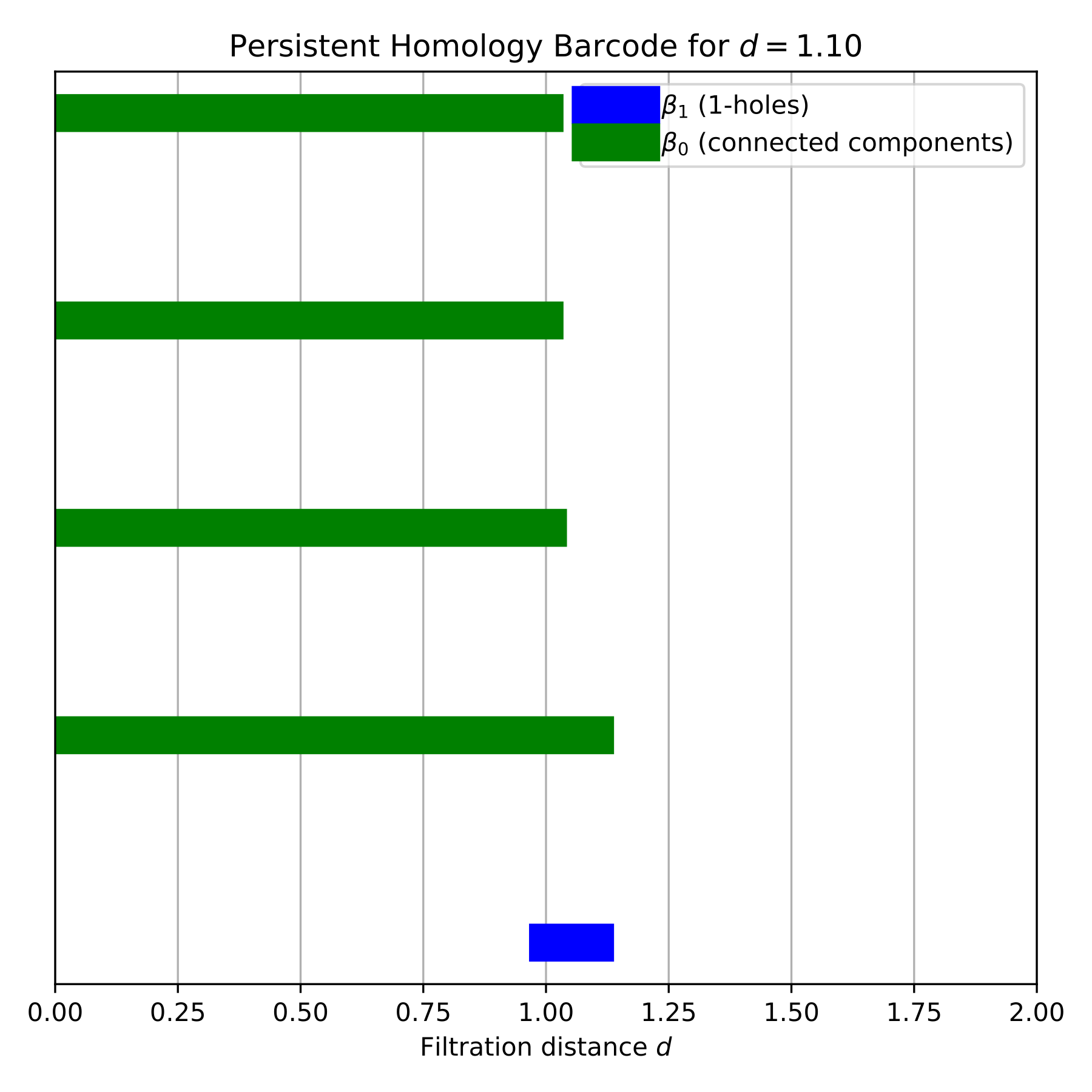

Supplement: Supplementary file 1 [file Data_Sheet_1.zip › TIFF/Figure_1e.tiff]

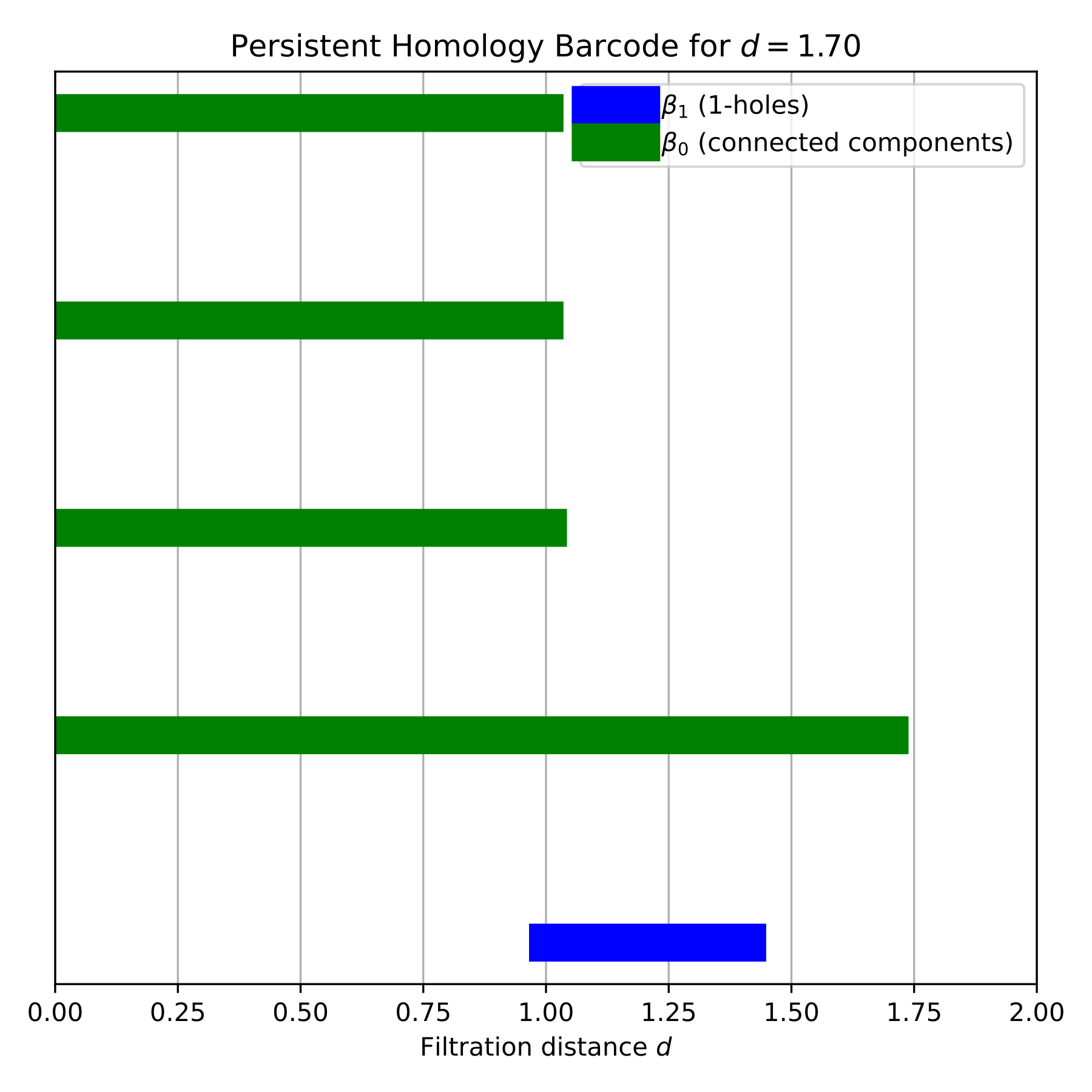

Supplement: Supplementary file 1 [file Data_Sheet_1.zip › TIFF/Figure_1f.tiff]

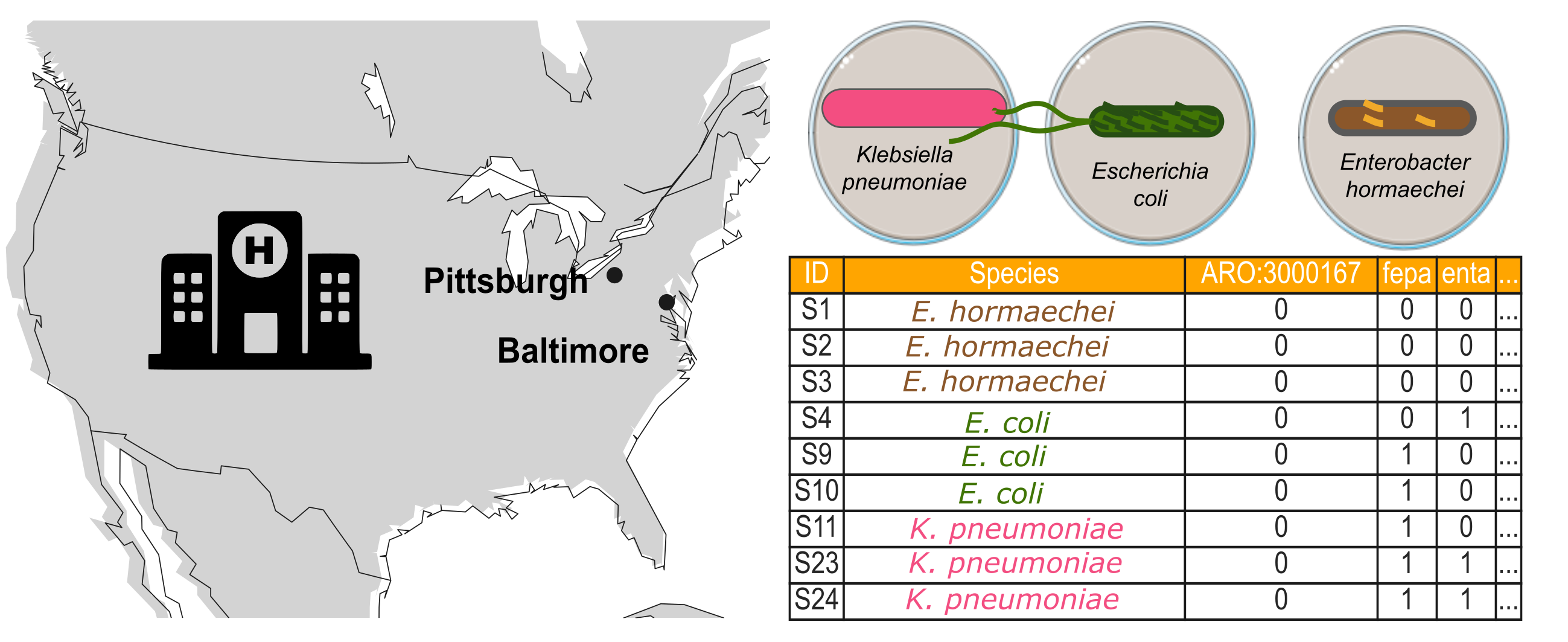

Supplement: Supplementary file 1 [file Data_Sheet_1.zip › TIFF/Figure_2.tiff]

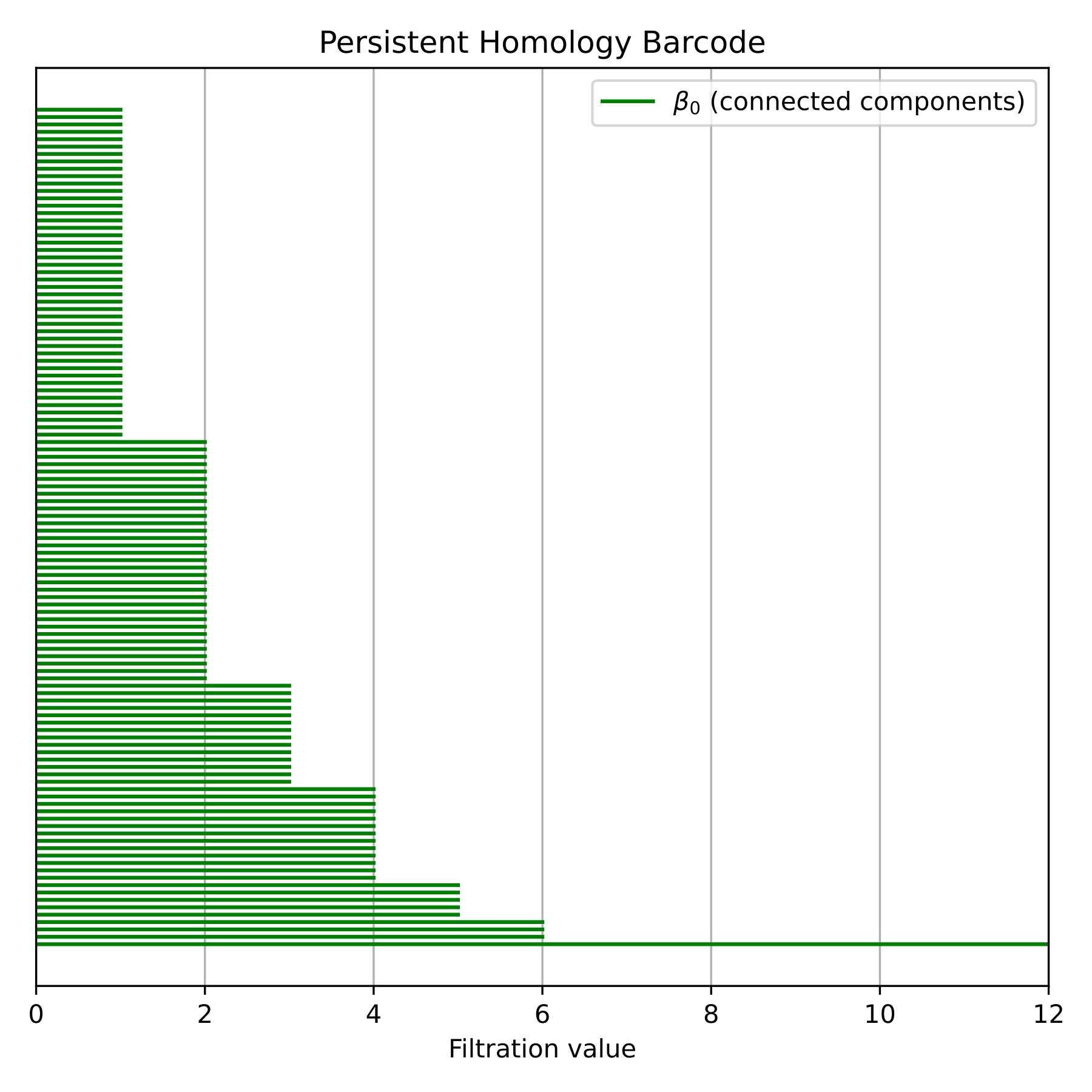

Supplement: Supplementary file 1 [file Data_Sheet_1.zip › TIFF/Figure_3a.tiff]

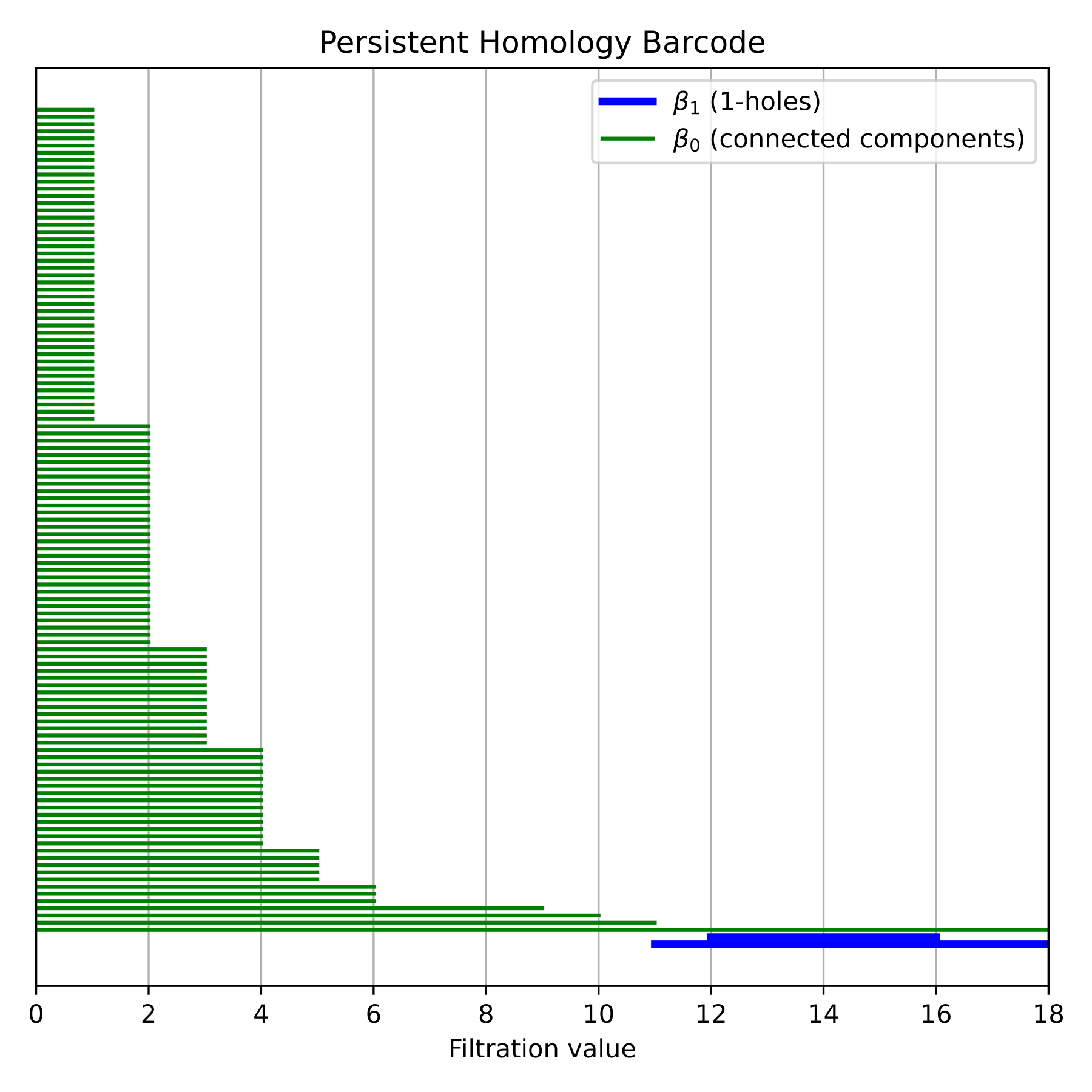

Supplement: Supplementary file 1 [file Data_Sheet_1.zip › TIFF/Figure_3b.tiff]

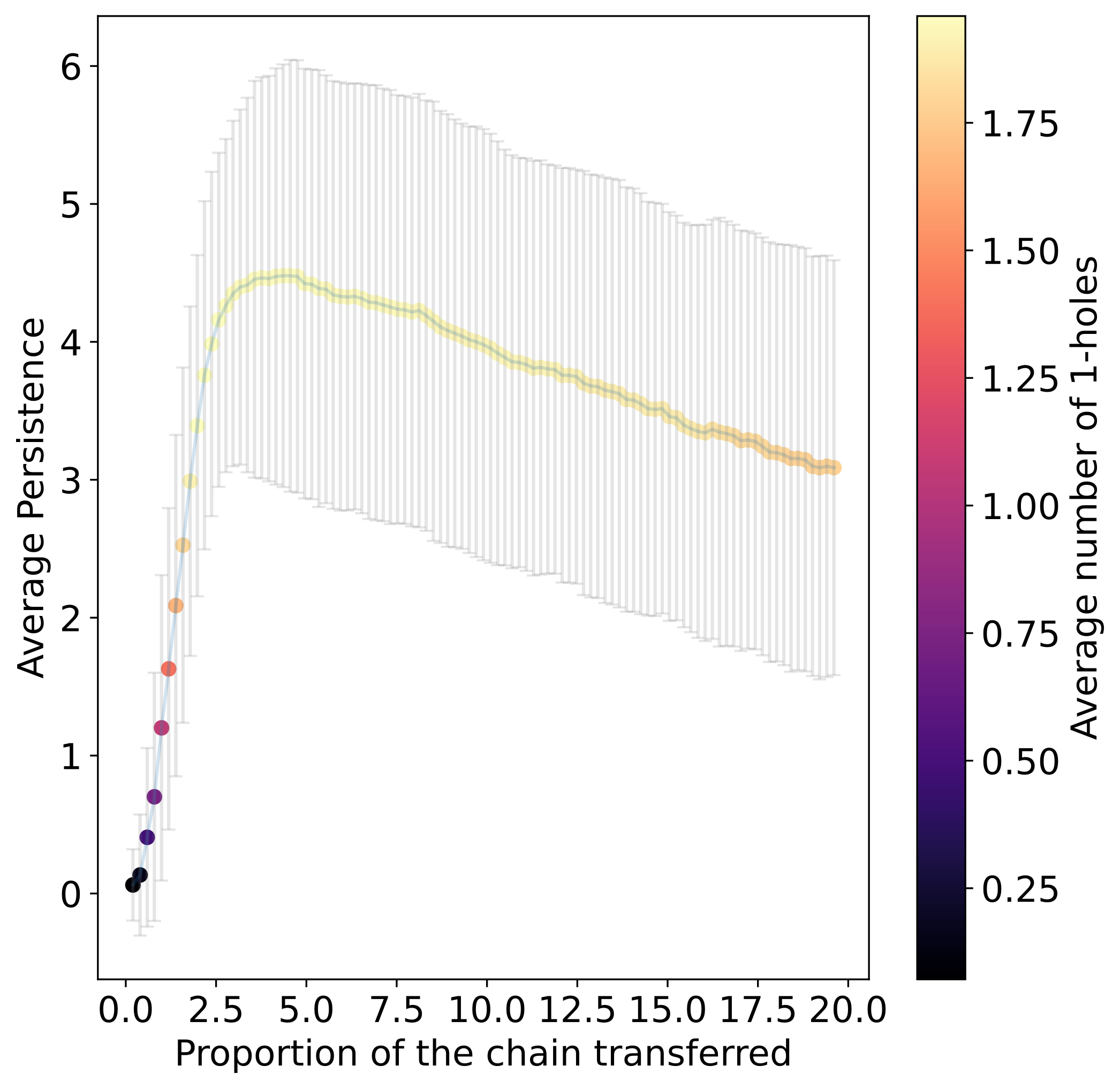

Supplement: Supplementary file 1 [file Data_Sheet_1.zip › TIFF/Figure_4a.tiff]

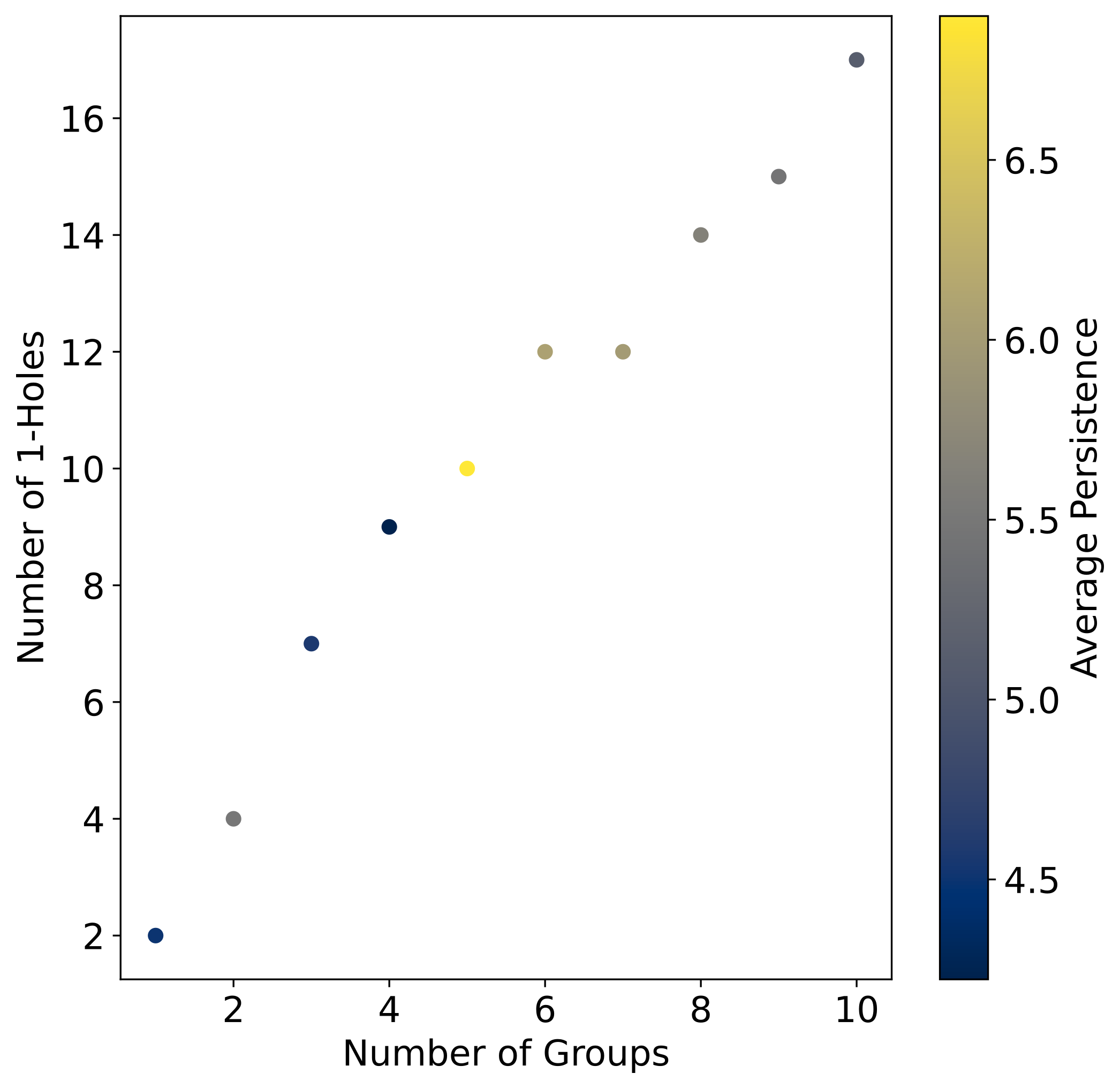

Supplement: Supplementary file 1 [file Data_Sheet_1.zip › TIFF/Figure_4b.tiff]

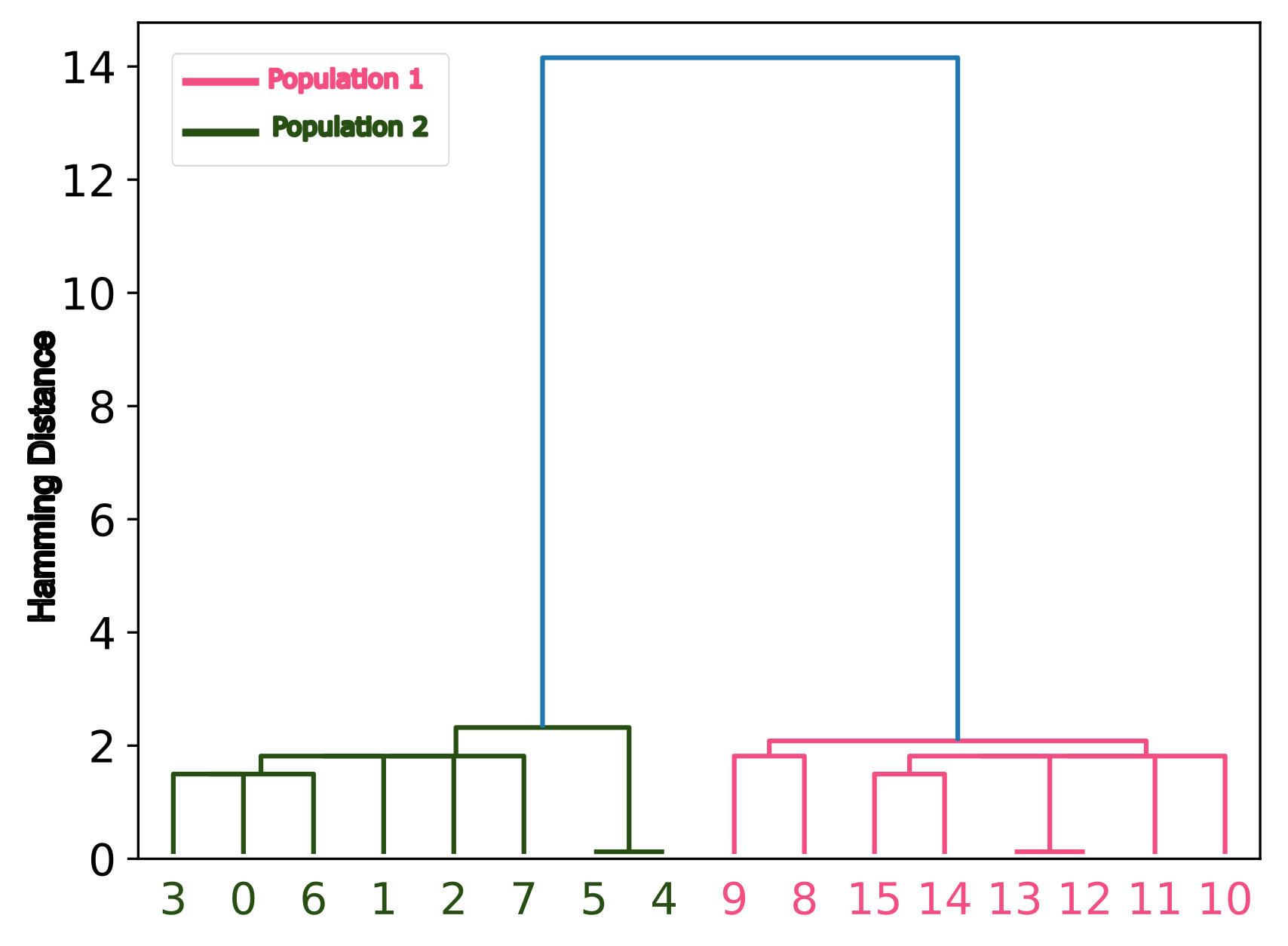

Supplement: Supplementary file 1 [file Data_Sheet_1.zip › TIFF/Figure_5a.tiff]

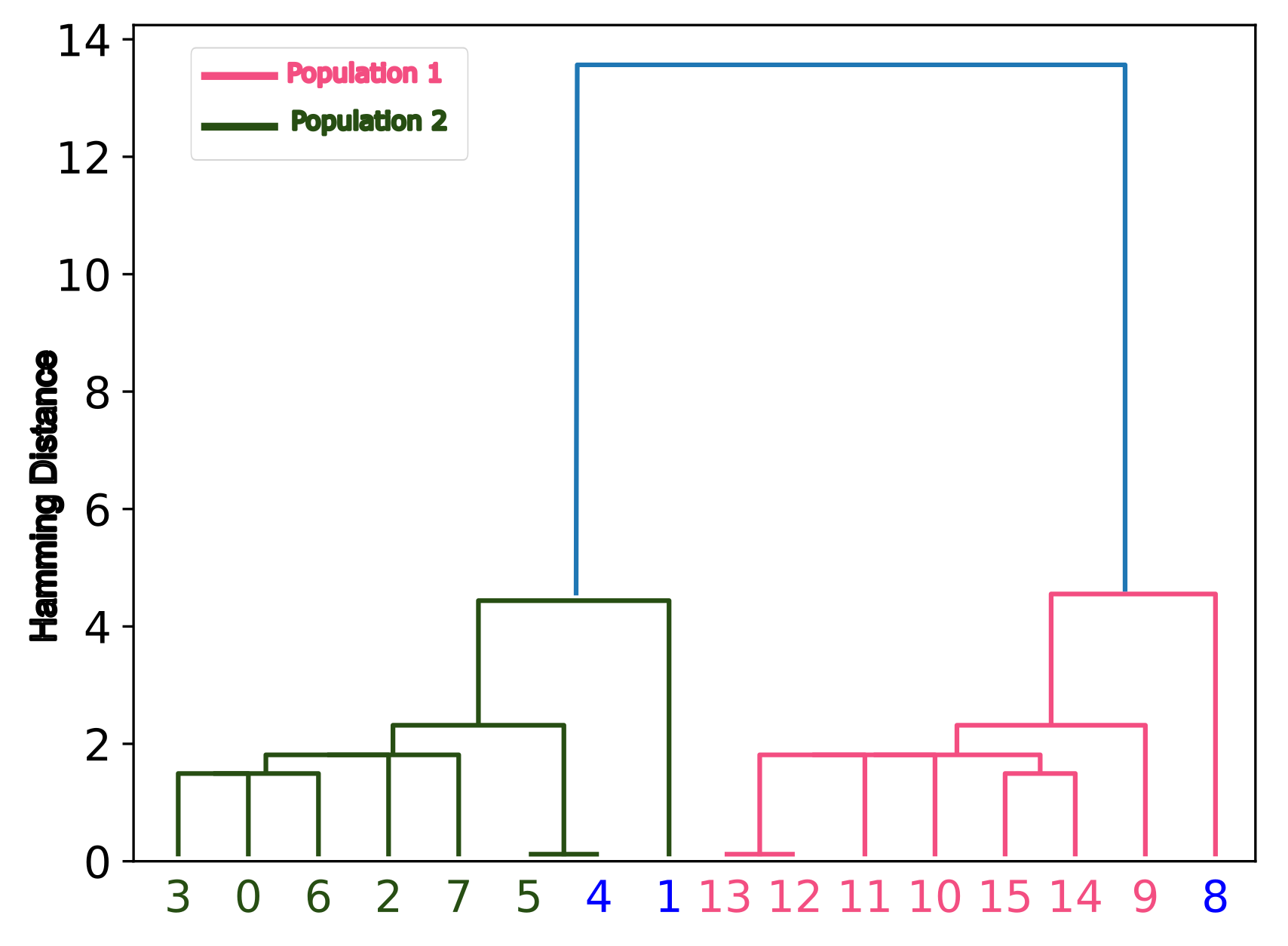

Supplement: Supplementary file 1 [file Data_Sheet_1.zip › TIFF/Figure_5b.tiff]

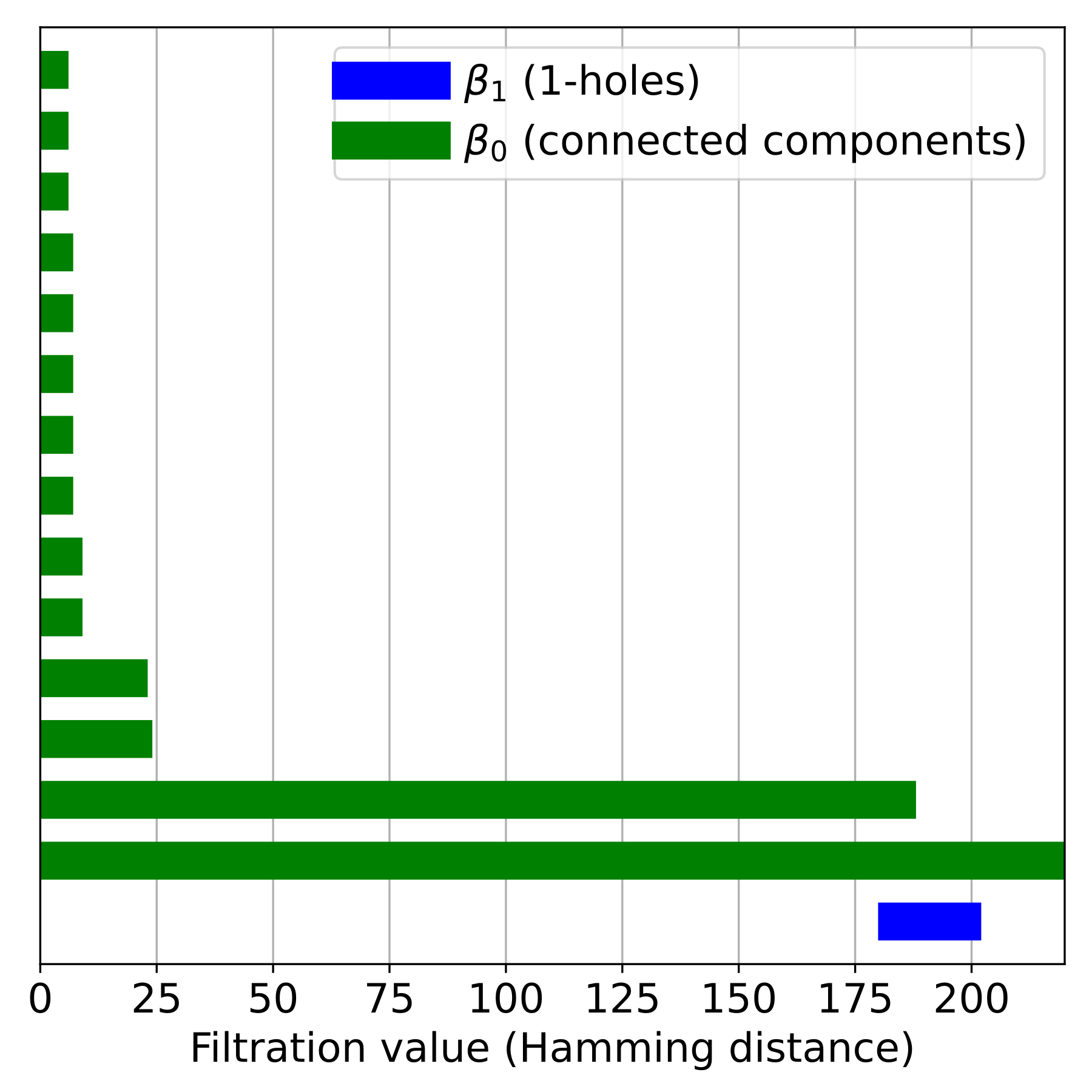

Supplement: Supplementary file 1 [file Data_Sheet_1.zip › TIFF/Figure_5c.tiff]

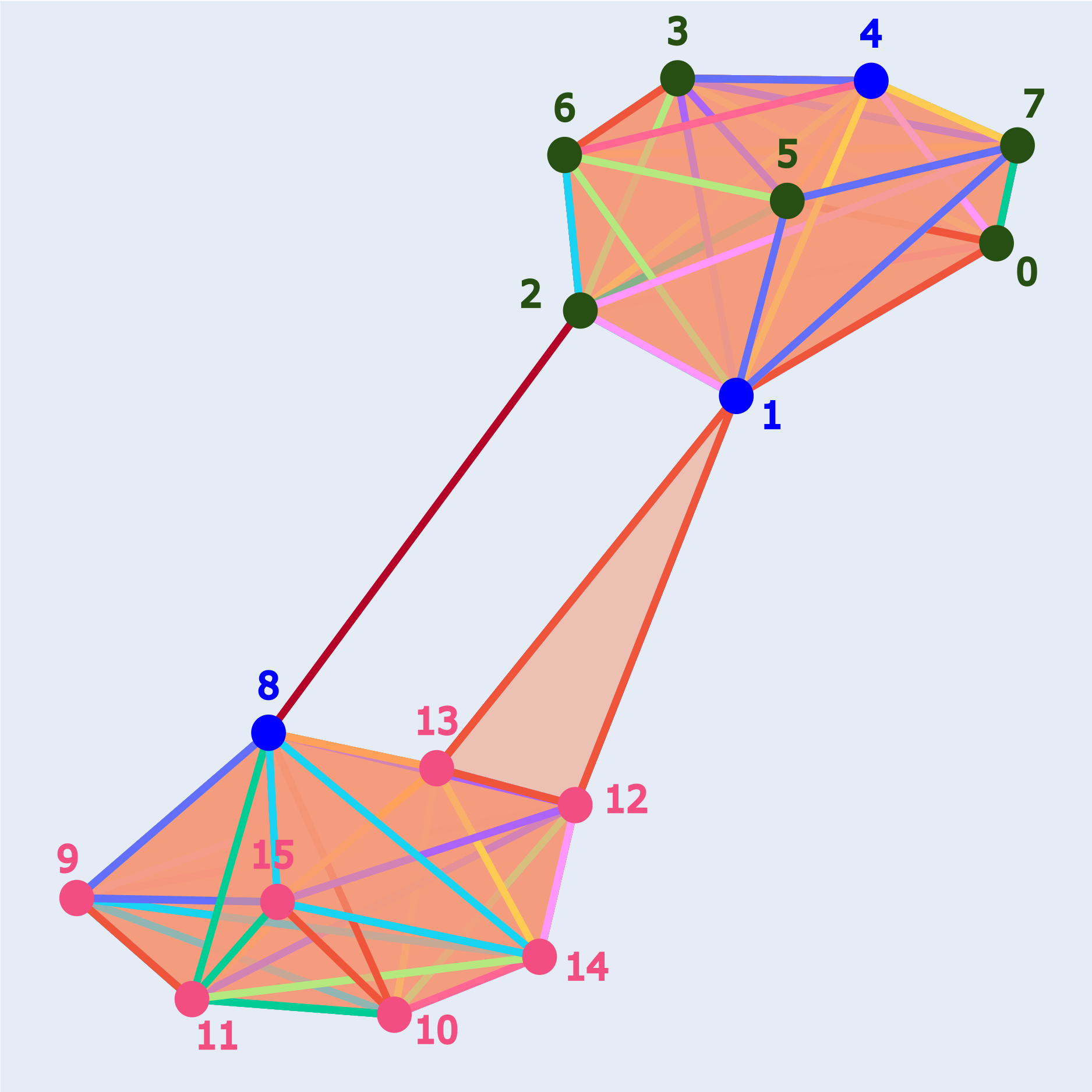

Supplement: Supplementary file 1 [file Data_Sheet_1.zip › TIFF/Figure_5d.tiff]

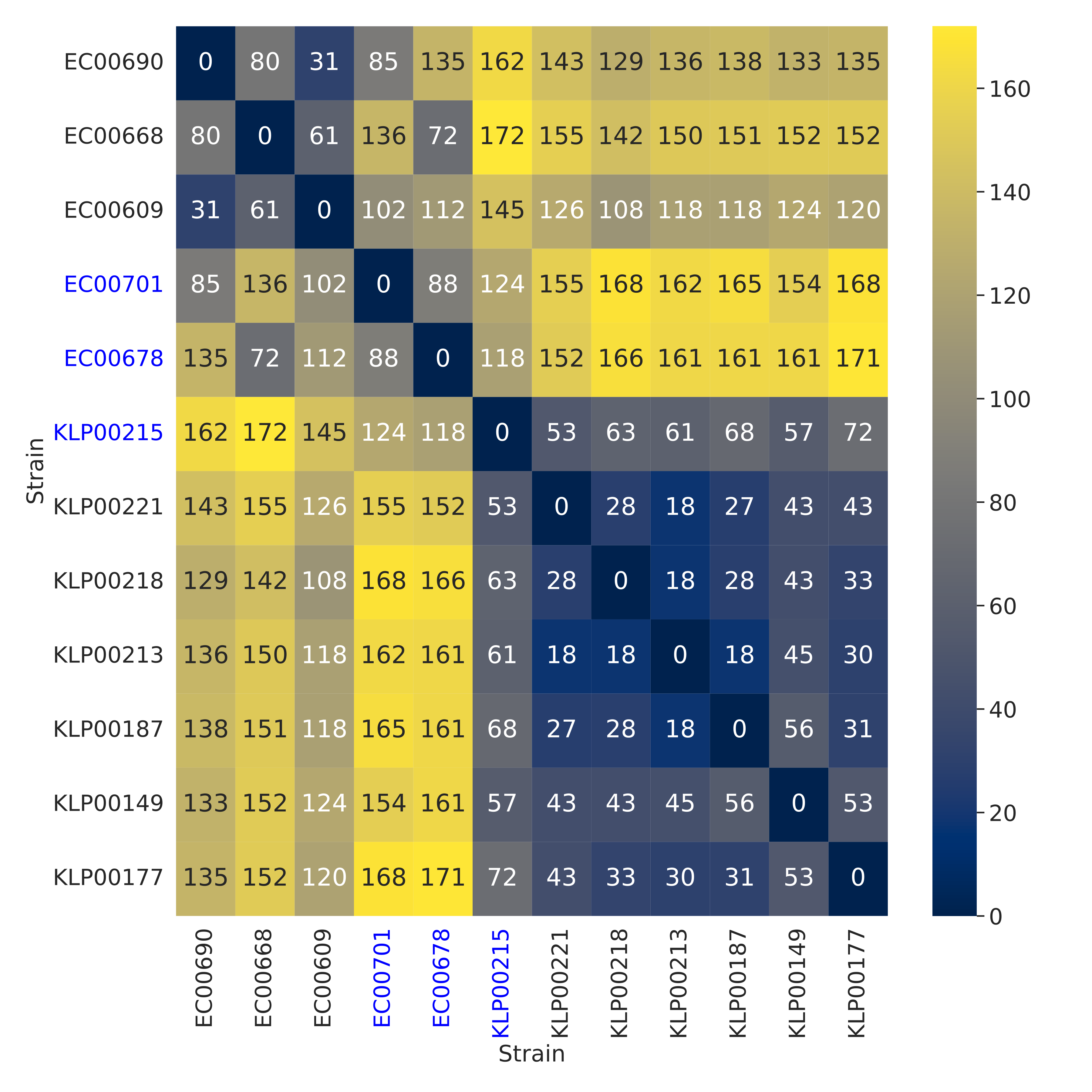

Supplement: Supplementary file 1 [file Data_Sheet_1.zip › TIFF/Figure_6a.tiff]

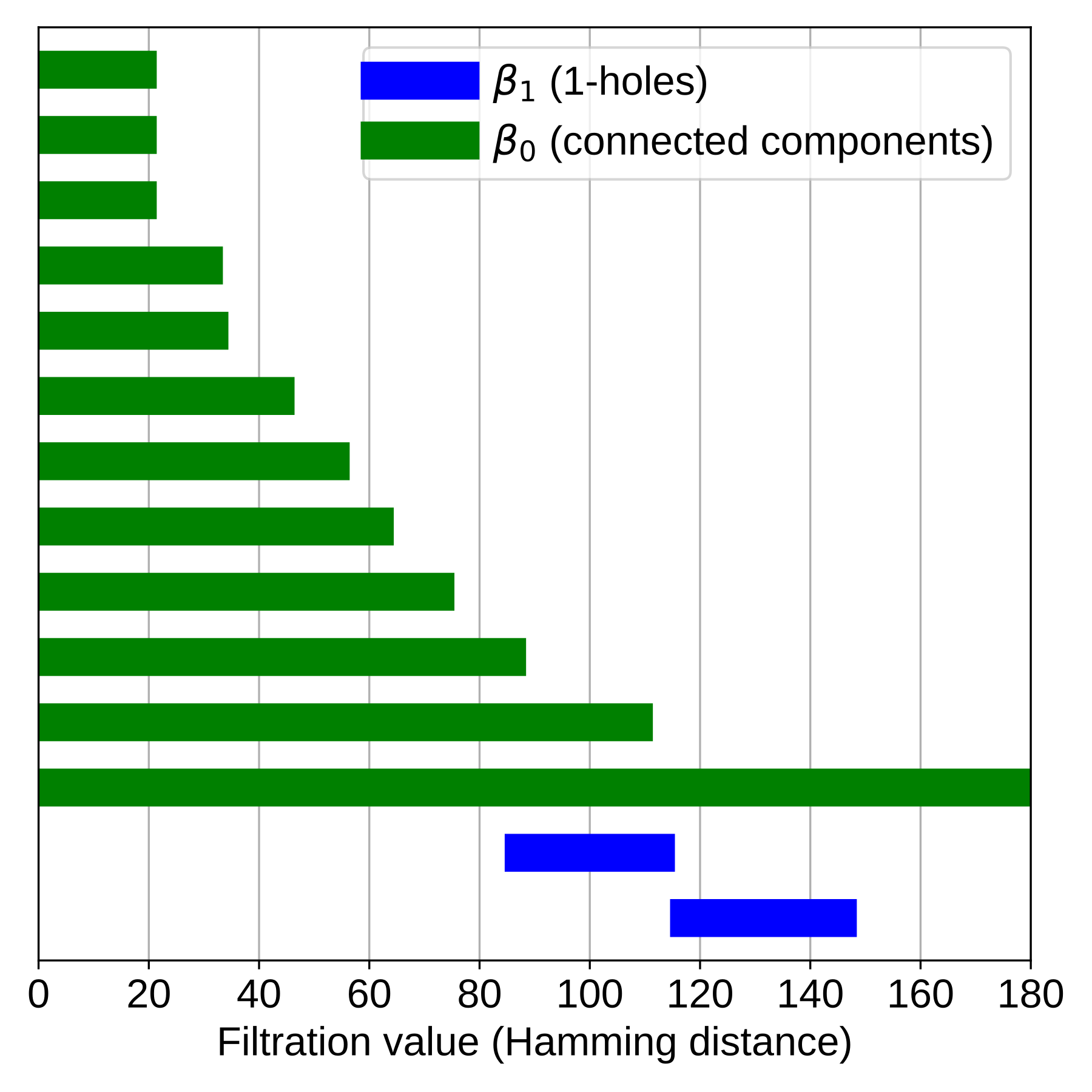

Supplement: Supplementary file 1 [file Data_Sheet_1.zip › TIFF/Figure_6b.tiff]

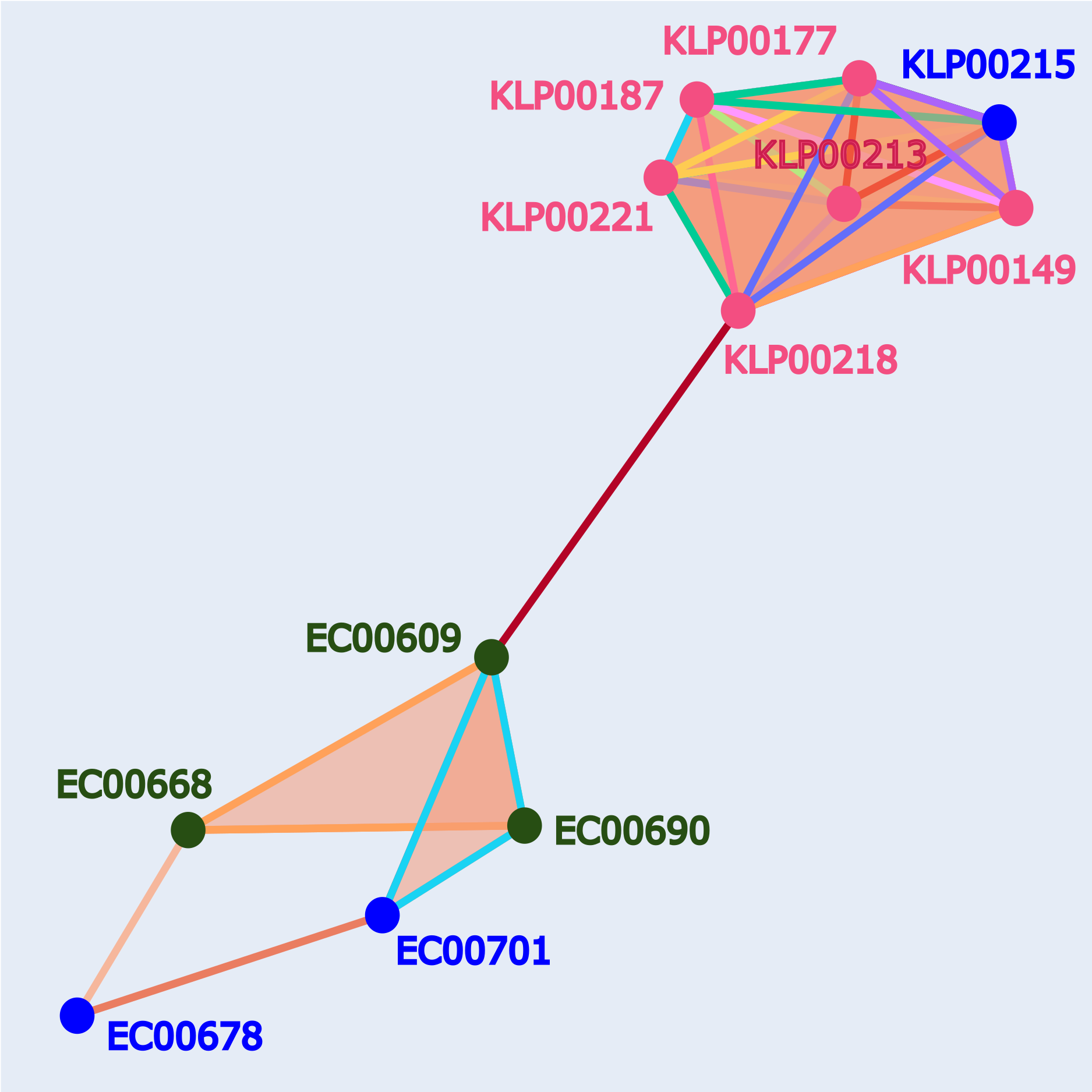

Supplement: Supplementary file 1 [file Data_Sheet_1.zip › TIFF/Figure_6c.tiff]

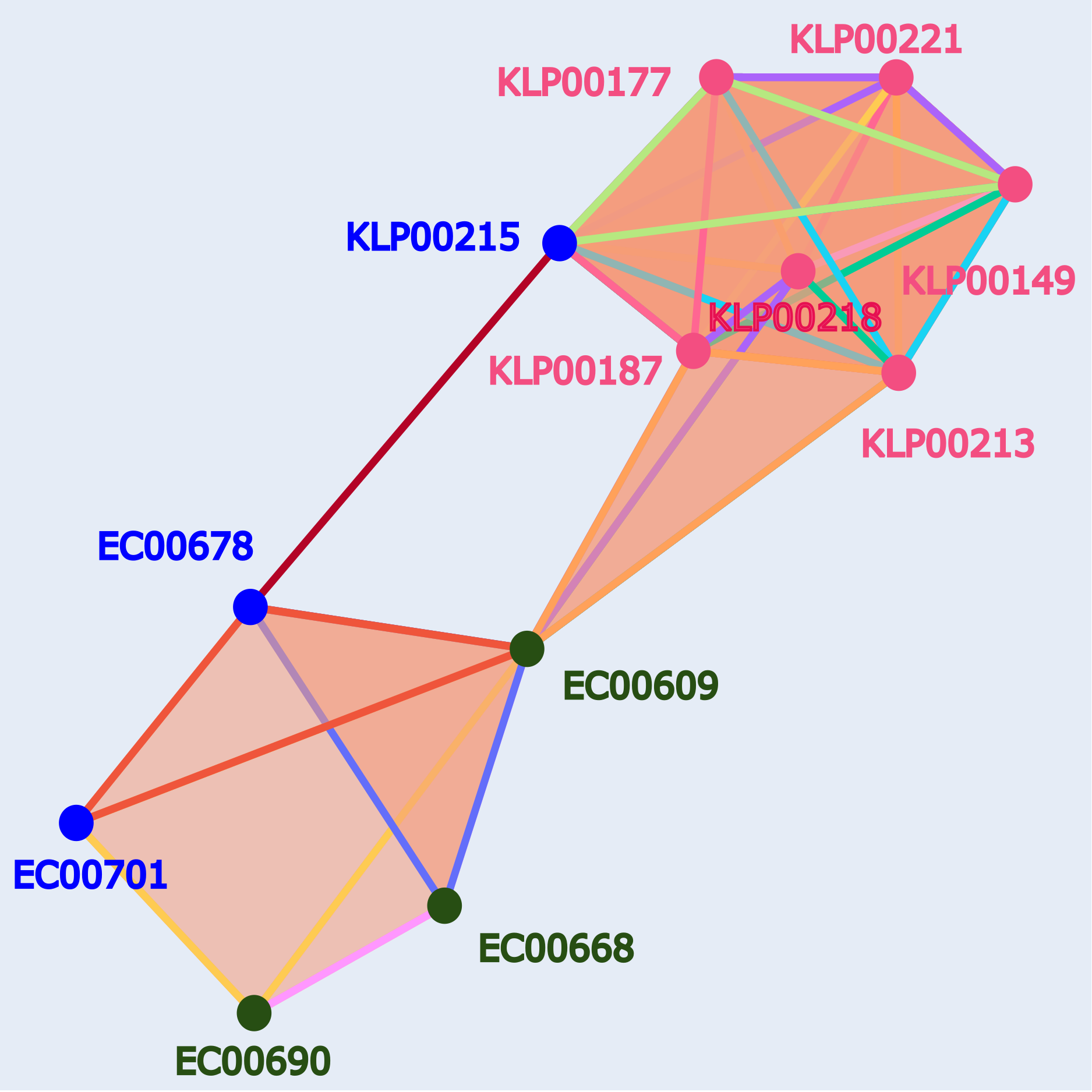

Supplement: Supplementary file 1 [file Data_Sheet_1.zip › TIFF/Figure_6d.tiff]

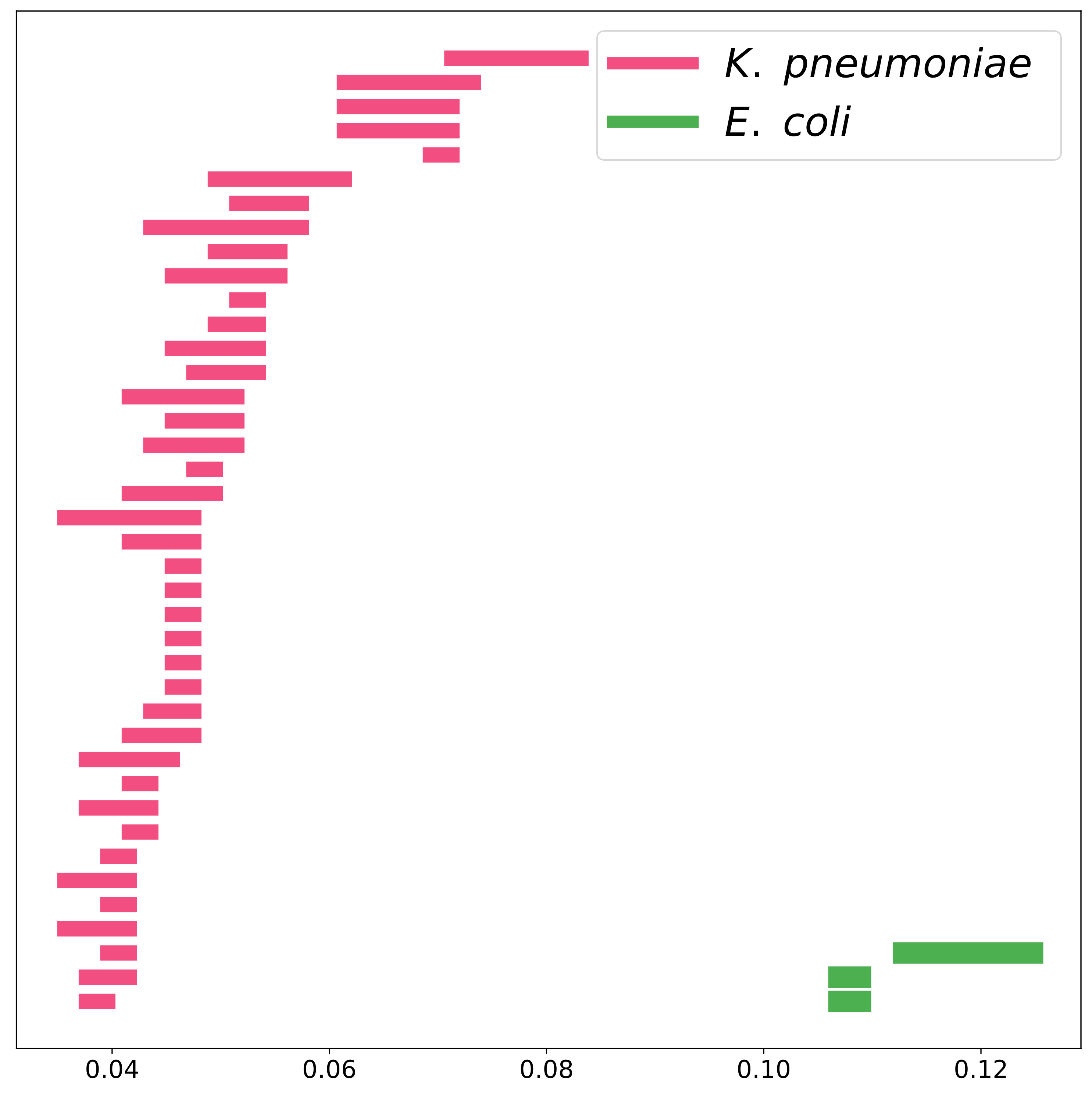

Supplement: Supplementary file 1 [file Data_Sheet_1.zip › TIFF/Figure_7a.tiff]

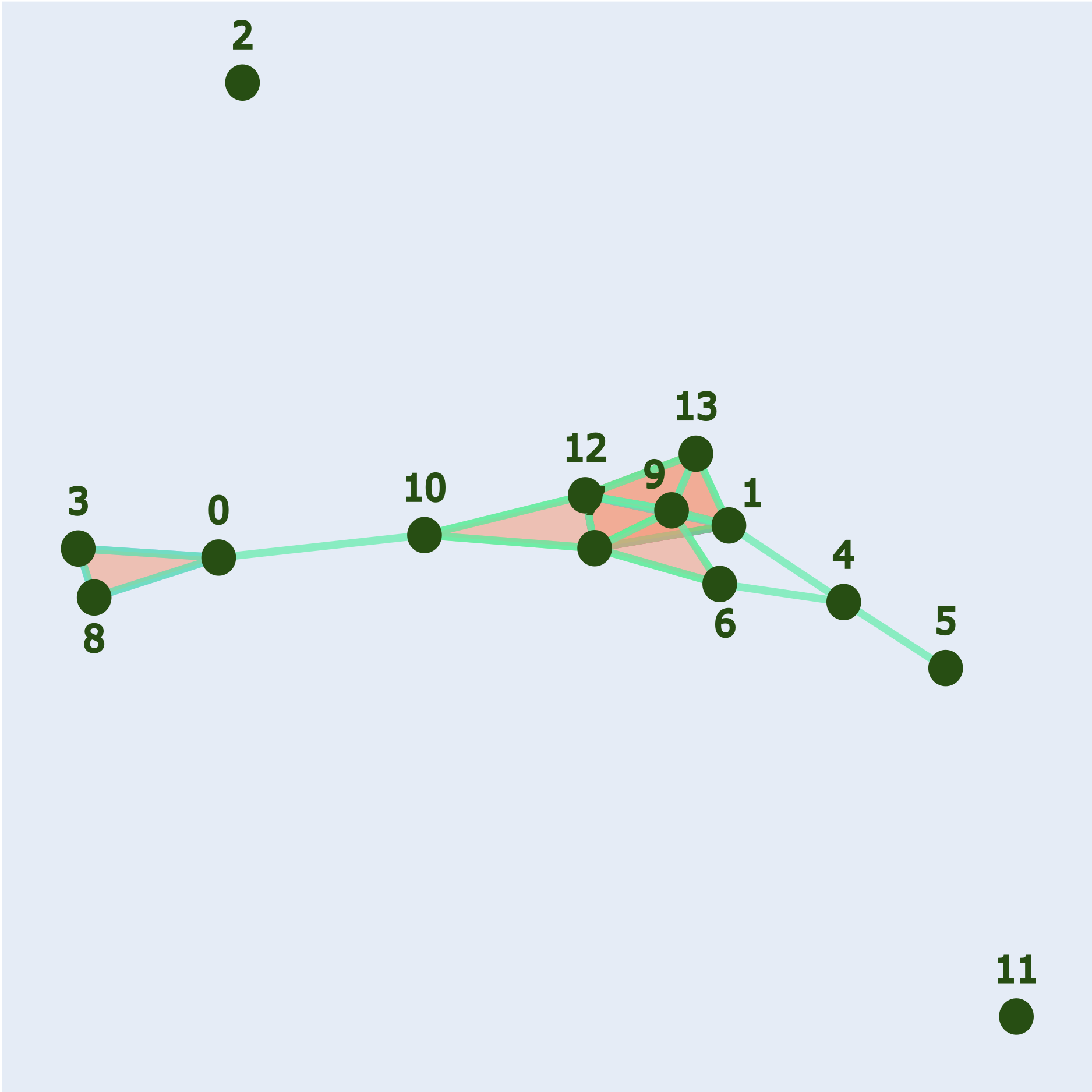

Supplement: Supplementary file 1 [file Data_Sheet_1.zip › TIFF/Figure_7b.tiff]

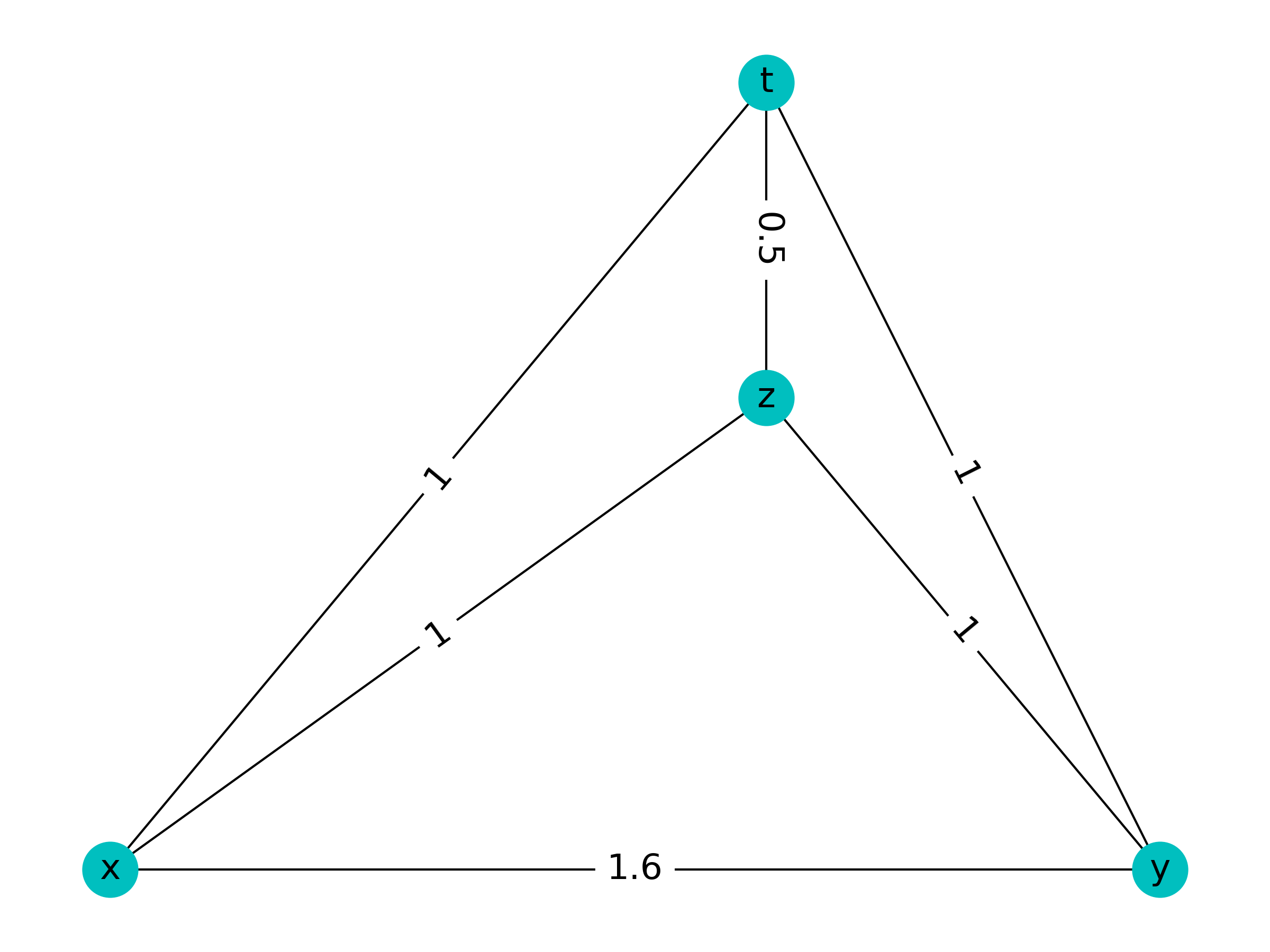

Supplement: Supplementary file 1 [file Data_Sheet_1.zip › TIFF/Figure_8.tiff]

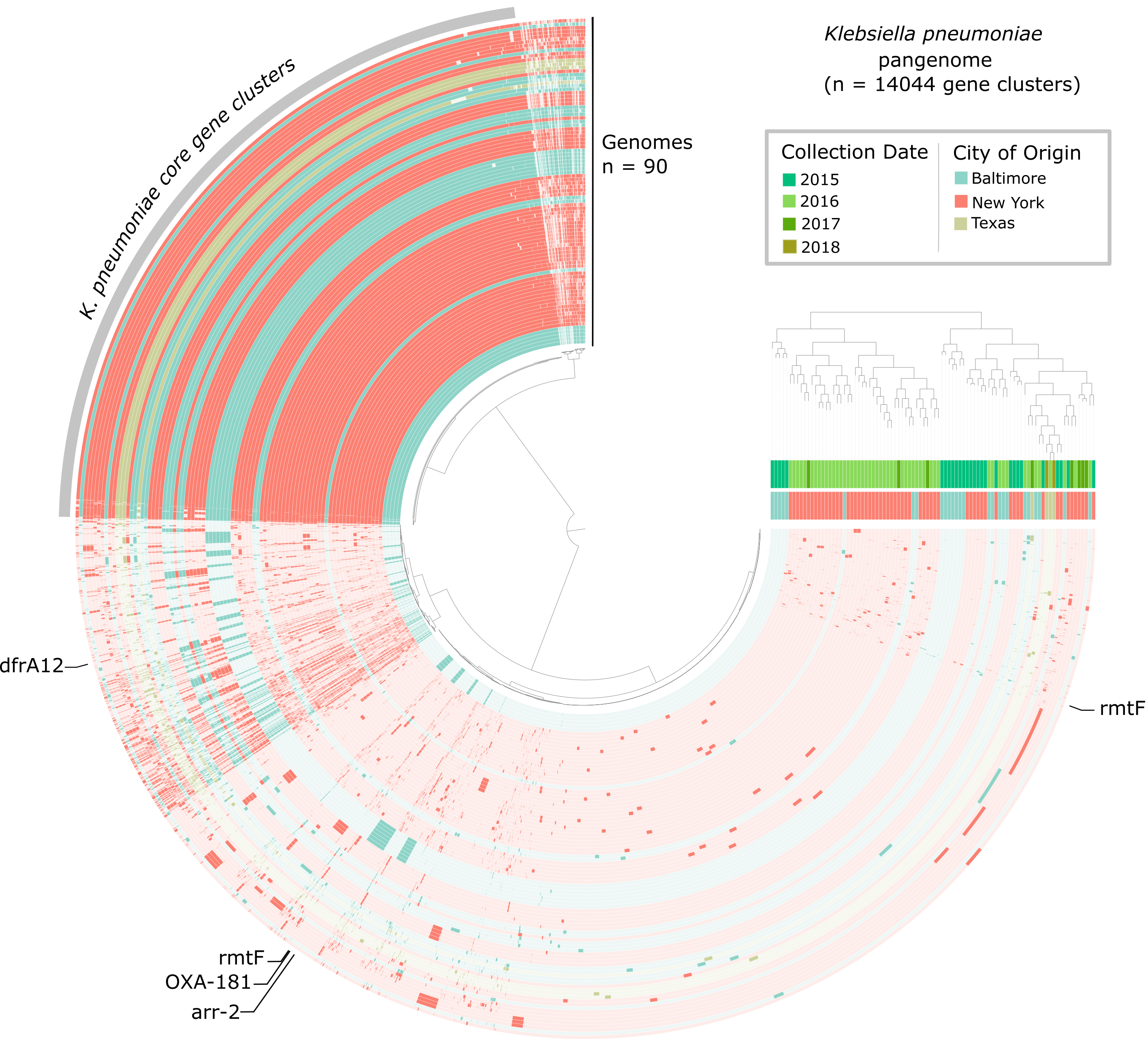

Supplement: Supplementary file 1 [file Data_Sheet_1.zip › TIFF/Figure_S1.tiff]
