# Supplementary material for: Topological data analysis captures horizontal gene transfer in antimicrobial resistance gene families among clinically relevant bacteria
Source: Front Microbiol. 2025 May 7;16:1461293. doi: 10.3389/fmicb.2025.1461293 (PMC12092391; doi:10.3389/fmicb.2025.1461293)
Supplement: Supplementary file 2 [file Data_Sheet_2.pdf]

Supplementary Material

1 PERSISTENT HOMOLOGY AND BETTI NUMBERS

Topological data analysis allows us to study the properties of a given data set by associating a form to measure similarity and then a simplicial complex that is a generalization of a graph. We will describe some preliminary concepts following (Dey and Wang, 2022), (Rabadán R., 2020), (Edelsbrunner and Harer, 2022)

Let $P = \{p_0, p_1, ..., p_k\}$ be a collection of points. A $k-$simplex generated by P will be denoted by σ_P, and it is the set of all points that satisfies

$$\sum_{i=0}^{k} \lambda_i p_i$$

where $\lambda_i \geq 0$ and $\sum \lambda_i = 1$. In particular, a $0-$simplex is a vertex, a $1-$simplex is an edge, and a $2-$simplex is a triangle. The dimension of a $k-$simplex will be k. A face of dimension k' of the simplex is an $k'-$simplex with a generated set of a nonempty subset of P.

A simplicial complex K will be a finite set of simplex that satisfies the following conditions:

1. If σ is a simplex of K, then every face of $sigma$ is in K.
2. For every $\sigma, \tau \in K$, the intersection $\sigma \cap \tau$ is an empty face or is a face of both simplices.

A $p-$chain in a simplicial complex K is a formal sum of the $p-$simplices σ_i with some coefficients. The $p-$chains with the $\mathbb{Z}_2$ additions form a group called the $p-$chain group, denoted by $C_p(K)$. Given a $p-$simplex $\sigma = \{v_0, v_1, ..., v_n\}$, the boundary of σ is

$$\partial_n \sigma = \sum_{i=0}^{n} (v_0, ..., \hat{v}_i, ...v_n),$$

where $\hat{v}_i$ denote that the vertex v_i is omitted. The boundary operator is a homomorphism $\partial_n : C_n(K) \longrightarrow C_{n-1}(K)$.

The boundary operator define two other groups, the $p-$th cycle group $\mathbf{Z}_p = \text{ker}\partial_p$ and the $(p-1)-$th boundary group $\mathbf{B}_{p-1} = \text{im}\partial_p$. Using homology, we can classify the cycles. For $p \geq 0$, the $p-$th homology group is the quotient group $H_p = \mathbf{Z}_p/\mathbf{B}_p$. If we take coefficients in $\mathbb{Z}_2$ then H_p is a vector space, and the dimension of such space is called the $p-$th Betti number $\beta_p = \text{dim}H_p$.

The Betti numbers allow us to understand the form and properties of the space we study. The Betti number β_0 counts the number of connected components, the Betti number β_1 tells us how many holes of dimension one we have, for example, if we consider only the perimeter of a circle or the circle without its interior, this would be a 1-hole.

Consider a finite simplicial complex K, and let $= K_0 \subset K_1 \subset \cdots \subset K_l = K$ be a finite sequence of nested sub complexes of K. The simplicial complex K, together with this sequence, is called a filtered simplicial complex.

Given a simplicial complex K and a filtration $= K_0 \subset K_1 \subset \cdots \subset K_l = K$, the inclusions $K_i \hookrightarrow K_j$ induces homomorphism between the corresponding homology groups $h_p^{i,j} : H_p(K_i) \longrightarrow H_p(K_j)$. The filtration also gives rise to a sequence of homomorphism $h_p^{i,j}$ induced for the inclusions, and with this we obtain the so-called homology module:

$$0 = H_p(K_0) \to H_p(K_1) \to \cdots \to H_p(K_i) \to \cdots \to H_p(K_j) \to \cdots \to H_p(K_n) = H_p(K).$$

The image of the homomorphism $h_p^{i,j}$ is called the $p-$th persistent homology group, and it is denoted by $H_p^{i,j}$. The dimension of such vector space is the $p-$th persistent Betti number. Each persistent homology group has all the information about when a homology class is born or dies. The information of when a class is born and die is captured in the barcodes or persistent diagram.

Consider an element $\gamma \in H_p(K_i)$. This element represents a p-dimensional feature in the filtration at time i. If γ does not exist for $j < i$, then we will say that the feature is born at time i, and we will say that it dies at time $l > i$ if it becomes zero in $H_p(K_l)$ or if its image in $H_p(K_l)$ coincides with the image of another class that was born earlier.

A barcode is a multiset of intervals of the form $[a, b) \in \mathbb{R}$ or $[a, \infty)$ that captures birth and death information from homology classes.

One of the most simplicial complexes used and implemented in topological data analysis is the Vietoris-Rips complex. Given a collection of points $X = \{x_i\} \in \mathbb{R}^n$, the Vietoris-Rips complex, denoted by $VR_t(X)$, is the simplicial complex where the vertices are elements of X and the $k-$simplices are those that satisfy d$(x_i, x_j) < r$ for every $1 \leq i, j \leq k$.

2 TOPOLOGY OF TREE-LIKE METRIC SPACES

2.1 tree-like spaces

A connected acyclic graph is called a tree. Tree are especially useful for modeling evolutionary phenomena. The edges of a tree are known as branches. Elements of trees are called their nodes. The grade one nodes are called leaf nodes.

All trees with nonnegative weights on their branches can induce a distance function between its nodes.

Definition 2.1. We say that a finite metric space $\mathcal{M}(M, d)$ is *tree-like* if there exists a tree $T(V, E)$ with non-negative weights on its edges and a function $\pi : M \to V$ that preserves distances. In other words,

$$d(x, y) = d_T(\pi(x), \pi(y)), \qquad \forall x, y \in M.$$

PROPOSITION 2.2. *Let M be a finite metric space, and let $m_0, m_1 \in M$ be two points at which the maximum distance is attained, i.e.,*

$$d(m_0, m_1) \geq d(m, m') \qquad \forall m, m' \in M.$$

Now, define $M_0 = M \setminus \{m_0\}$ and $M_1 = M \setminus \{m_1\}$ as metric spaces. Then, for each $r < d(m_0, m_1)$, we have

$$V(M, r) = V(M_0, r) \cup V(M_1, r).$$

Moreover, $V(M_0, r) \cap V(M_1, r) = V(M_0 \cap M_1, r)$*, where* $M_0 \cap M_1 = M \setminus \{m_0, m_1\}$.

PROOF. The result follows easily from the observation that no simplex in $V(M, r)$ contains both m_0 and m_1 for all $r < d(m_0, m_1)$. $\square$

On the other hand, it is easy to see that if we have a finite metric space M and a distance $r > 0$, we can define an equivalence relation between the points of M if they are in the same connected component of the r-neighborhood graph of M. This relation can also be defined on the complete graph induced by the distance matrix; in this case, two points are said to be equivalent if there exists an r-path between them. We will use this same notation to refer to the equivalence relation defined by r-paths between the nodes of $V(M, r)$, which, in turn, corresponds to the nodes of the different connected components. That is, the partition of the metric space M defined by the equivalence relation of r-paths $\simeq_r$, $M = \bigsqcup_\alpha M_\alpha$, allows us to express the complex $V(M, r)$ in terms of the complexes that constitute each of its connected components:

$$V(M, r) = \coprod_\alpha V((M_\alpha, d_M|_{M_\alpha \times M_\alpha}), r).$$

PROPOSITION 2.3. *The resulting subspaces obtained by removing leaves from an additive space are also additive.*

THEOREM 2.4. *Let* M *be an additive metric space, and* $r \geq 0$*. Then the simplicial complex* $V(M, r)$ *is a disjoint union of acyclic complexes, and therefore,*

$$H_i(M) = \{0\} \qquad \forall i \geq 1.$$

PROOF. It is clear that the result holds for all r greater than or equal to the maximum distance in M, as the resulting complex is homotopic to a ball of dimension $|M| - 1$.

For the case where r is less than the greatest distance, we proceed by induction on the cardinality of the metric spaces. We will consider as the induction hypothesis that the proposition is true for all metric spaces with cardinality less than n. We can see that the result holds for the base case, $|M| = 2$, since in the Vietoris-Rips complex $V(M, r)$, each of the points constitutes a connected component.

We also saw that one way to observe the connected components of $V(M, r)$ is by considering the equivalence relation $\simeq_r$ given by the r-paths. Thus, using the same notation, if $\mathbf{C} = \{M_\alpha\}_{\alpha \in \Lambda}$ is the family of equivalence classes, then we have that

$$V(M, r) = \coprod_{\alpha \in \Lambda} V((M_\alpha, d_M \mid_{M_\alpha \times M_\alpha}), r).$$

STEP 1. INDUCTION

Now, if $V(M, r)$ is not connected, we can assert that each subset in the partition has a cardinality smaller than that of M. Therefore, and due to Proposition **??**, we know that each of these subspaces is, in turn, an additive metric space. Thus, the result holds through the induction hypothesis.

Let's assume that $V(M, r)$ is connected. Take m_0 and m_1 in M as two points that maximize the distance in M. Consider $M_0 = M \setminus \{m_0\}$ and $M_1 = M \setminus \{m_1\}$ as metric spaces. According to Proposition 2.2,

we know that

$$V(M,r) = V(M_0,r) \cup V(M_1,r),$$

and that $V(M_0,r) \cap V(M_1,r) = V(M_0 \cap M_1, r)$, where $M_0 \cap M_1 = M \setminus \{m_0, m_1\}$.

Here, we can observe that both M_0, M_1, and their intersection are additive metric spaces with a cardinality smaller than that of M. Thus, by the induction hypothesis, we have that $V(M_0,r)$, $V(M_1,r)$, and $C(M_0 \cap M_1, r)$ constitute a disjoint union of acyclic complexes.

In conclusion, and starting from Proposition **??**, it is sufficient to prove that $V(M_0,r)$, $V(M_1,r)$, and $V(M_0 \cap M_1, r)$ are connected.

STEP 2. M_0 IS CONNECTED: ADDITIONAL NOTATION

To prove that $V(M_0,r)$ is connected, it suffices to demonstrate that an r-path exists between any two elements of M_0. By Lemma **??**, we can assume that M is included in $\mathcal{M}(T)$ for some additive tree T in such a way that the leaves of T are contained in the image of M. We will use this structure to prove what we want, although it will require introducing additional notation.

Let's start by observing that for each leaf e in the tree, there is a unique junction $j(e)$ closest to it, unless there are no junctions at all. In the latter case, the tree is a line with intermediate nodes, and the result is immediately obtained. Let's assume, therefore, that there is at least one junction in T.

For each pair (v,e) where $v \in V_T$ and e is an edge of T containing v, $Br(e,v)$ will denote the subtree formed by the vertices $v' \in V_T$ such that the minimal path from v to v' passes through e. We will also refer to it as the *branch of* T *passing through* v *and* e.

Now, let's consider the nodes connected to the nearest junction of m_0. That is, take the nodes $\{v_0, v_1, \ldots, v_n\}$ such that $(j(m_0), v_i) \in E_T$ and define $\mathcal{B}_i = Br(j(m_0), (j(m_0), v_i))$ for each of them. Without loss of generality, we can assume that $m_0 \in \mathcal{B}_0$ and $m_1 \in \mathcal{B}_1$.

Let's also observe that:

$$d(v, j(m_0)) \leq d(m_0, j(m_0)) \quad \forall v \in \mathcal{B}_i, \quad i \geq 2.$$

Otherwise, it contradicts the maximality of the distance from m_0 to m_1. As a consequence of this inequality, we have that:

$$d(w,v) \leq d(w,m_0) \quad \text{where } v \in \mathcal{B}_i, \quad i \geq 2 \text{ and } w \in \mathcal{B}_j, \quad j \geq 1.$$

Indeed, let w and v be as described before, and consider the path in T from w to v passing through $j(m_0)$. This path is obtained by concatenating the minimal paths from w to $j(m_0)$ and from $j(m_0)$ to v. Because the distance between u and w is defined by the length of the minimal path between them, the length of the path we just constructed may be greater than this distance. That is,

$$d(u,w) \leq d(w, j(m_0)) + d(j(m_0), v). \tag{S1}$$

Using the previous inequality, we can then conclude that

$$d(u,w) \leq d(w, j(m_0)) + d(j(m_0), v) \leq d(w, j(m_0)) + d(j(m_0), m_0) = d(w, m_0).$$

STEP 3. M_0 IS CONNECTED BY r-PATHS

Let's now prove that $V(M_0, r)$ is connected by r-paths. Let $m, m' \in M_0$. Since M is r-connected, there exists an r-path $(x_0, x_1, \ldots, x_n)$ that connects them. If m_0 is not in this path, then the points are connected by a path in $V(M_0, r)$. Let's assume that $x_i = m_0$ for some i; to simplify the process, we can assume that the path passes through m_0 exactly once. In particular, we have $d(x_{i-1}, m_0) \leq r$ and $d(m_0, x_{i+1}) \leq r$. Now we will proceed by cases.

First, let's assume that $M \cap (\mathcal{B}_0 \setminus \{m_0\})$ is non-empty. Let $\overline{m}$ be the closest point to m_0. It is clear that by replacing the segment v_{i-1}, m_0, v_{i+1} with $v_{i-1}, \overline{m}, v_{i+1}$, we obtain an r-path that does not pass through m_0, which is precisely what we were looking for.

Otherwise, when $M \cap (\mathcal{B}_0 \setminus \{m_0\})$ is empty, we select any leaf $m^* \in \mathcal{B}_i$ for some $i \geq 2$. By inequality S1, we see that we can replace the segment v_{i-1}, m_0, v_{i+1} with v_{i-1}, m^*, v_{i+1}, thereby obtaining an r-path entirely contained in $V(M_0, r)$. This concludes that $V(M_0, r)$ is connected by r-paths.

The result for M_1 and $M_0 \cap M_1$ is achieved in the same way, as we can independently remove occurrences of m_0 and m_1 in the r-paths. This completes the proof. □

The proof presented here follows the general logic of the article but also fills in the gaps left for the reader and introduces a couple of differences compared to the original. Specifically, these differences lie in the formulation of the induction and the simplification of paths containing m_0.

As can be naturally inferred, persistent homology allows us to extend this result to describe the consequences of the additivity of M in the simplicial complexes associated with it, without limiting ourselves to a particular r.

THEOREM 2.5. *Given an additive metric space M, the p-level persistence diagram of $V(M, r)$ is empty for all $p > 0$.*

This is how persistent homology allows us to discern when we can use a phylogenetic tree to explain a sample's evolutionary process.

3 DEVELOPMENT OF FUNCTIONS FOR THE USE OF TDA IN BACTERIAL EVOLUTIONARY RELATIONSHIPS

Were conducted using Python 3.7 and the Gudhi package Maria et al. (2014). We developed custom functions for various computations:

- `calculate_hamming_matrix`: This function generates a distance matrix between genomes from a presence-absence gene matrix. It utilizes the `hamming` function from the `scipy.spatial.distance` library.
- `create_complex`: This function takes a distance matrix as input and constructs a Vietoris-Rips simplicial complex using the `RipsComplex` function. It computes the simplex tree of dimension 3 using `create_simplex_tree` and calculates homological persistence with `persistence`, all imported from the `Gudhi` package for Python.
- `visualize_simplicial_complex`: This function graphically represents the simplicial complex for a given filtration level, utilizing `networkx`.

- To visualize persistence diagrams and barcodes, we used `plot_persistence_diagram` and `plot_persistence_barcode` functions.

These tools and functions enabled a comprehensive analysis of genomic data, facilitating insights into our dataset's complex relationships and structures.

4 ANTIBIOTIC PROFILES IN BALTIMORE CAMDA DATA

Antibiotic resistance profiles were characterized using the Resistance Gene Identifier (RGI) 6.0.2 and the Comprehensive Antibiotic Resistance Database (CARD) 3.2.6 (Alcock et al., 2022) as reference. Reads and contigs of interest (i.e., those which were assigned as *Enterobacter*, *Escherichia*, *Klebsiella*, or a subtaxon of any of these) were extracted by the use of KrakenTools 1.2 (Lu et al., 2022). The extracted reads were later passed through another assembly with MEGAHIT. RGI was executed on complete (sample- and city-level) assemblies and extractions.

To carry out antibiotic resistance predictions, assemblies of the reads were used. Combinations were made to obtain the assemblies by extracting the reads corresponding to each species and then assembling or reversing this process. Additionally, to increase the likelihood of assemblies, all samples from the same city were mixed, and then the reads were assembled. As a result, we have the following processes.

1. Coassemblies: Take all reads of each city and assemble them by city.
2. ExtraxtedCoassemblies: From the assemblies gotten by Coassemblies, extract *Escherichia*, *Klebsiella*, and *Enterobacter* sequences
3. ExtractedReadAssemblies: Take the reads (before the assembling), extract the *Escherichia*, *Klebsiella*, and *Enterobacter* sequences, and then assemble them by city.

However, of the 505 AMR markers, only 180 had an ARO number associated, i.e., could be predicted in metagenomes using CARD. The remaining 325 markers were obtained from NCBI, constituting our manually curated database. BLASTn aligned contigs from metagenomes against this manually curated database, and matches were marked as a presence in the table. Two types of contigs were considered by varying first assembling and then extracting or vice versa (Coassembled reads Extracted and ExtractedRead-Assembled).

5 PANGENOME ANALYSIS

Pangenome analysis was inspired by the pipelines of Dutter et al. (Utter et al., 2020) and Delmont et al. (Delmont and Eren, 2018). *Klebsiella pneumoniae* genomes (n=90) were downloaded from NCBI RefSeq and sequenced between 2015 and 2018 in the U.S. cities of Baltimore, New York, and Texas. Then,`anvi-script-reformat-fasta` streamline contig genome names.

Anvi'o (version 8) (Eren et al., 2015) created a genome database via `anvi-gen-contigs-db`. Bacterial single-copy core genes were identified in each genome using `anvi-run-hmms`, while open reading frames (ORFs) were identified via Prodigal (V2.6.3) (Hyatt et al., 2010). The `anvi-get-sequences-for-gene-calls` command facilitated the export of ORFs, followed by functional annotation with InterProScan (V5.64-96)(Jones et al., 2014), which includes TIGRFAM(Haft et al., 2001), Pfam(El-Gebali et al., 2019), SUPERFAMILY(Wilson et al., 2009), and Gene3D(Lees et al., 2012). Antimicrobial resistance (AMR) genes were annotated through RGI with the Comprehensive Antibiotic Resistance Database (CARD) (Alcock et al., 2022). `anvi-import-functions` integrated

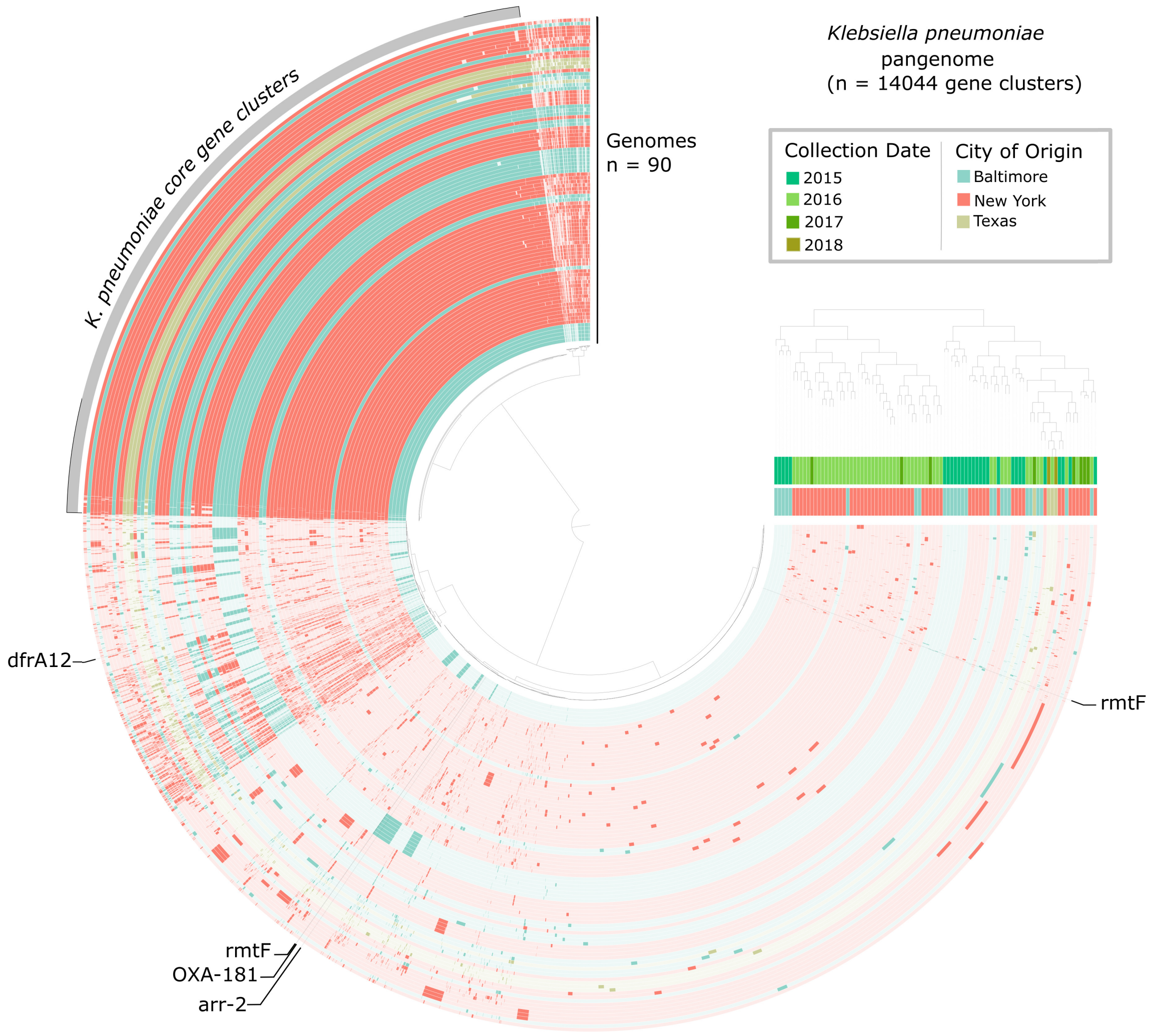

Figure 1: Resistance markers in the Pangenome of *K. pneumoniae*. Samples of *K. pneumoniae* from Baltimore, New York, and Texas were used, collected between 2015 and 2018. The resistance markers predominantly present in Baltimore are shown in the figure.

InterProScan and CARD annotations into the database. NCBI COG annotations were also added for broader functional categorization with `anvi-run-ncbi-cogs` utilizing `blastp` for searching.

Pangenome was calculated with `anvi-pan-genome` with the flag `--use-ncbi-blast` (Altschul et al., 1990) and a parameter `--mcl-inflation 10` (Van Dongen and Abreu-Goodger, 2012) to identify groups of genes. Function enrichment and their frequencies were obtained by grouping genomes according to the city in which they were isolated. For this step, `anvi-get-enriched-functions-per-pan-group` (Shaiber et al., 2020) was used, specifying the annotation source as CARD. Finally, the distribution of gene clusters in the pangenome highlighted unique AMRs of Baltimore and was visualized using `anvi-display-pan`.

5.1 *Klebsiella pneumoniae* pangenome shows AMRs unique in Baltimore

Pangenome (Figure 1)was constructed using genomic data from 90 isolates collected from different cities in the United States. Of these, 60 genomes were isolated from New York (NYC), 26 from Baltimore (BAL), and four from Texas (TX). The analysis revealed 14,044 gene clusters containing a total of 496,576

genes. The pangenome contained 3,598 core genes cluster, representing 25.6% of the total pangenome, and 3,936 accessory genes cluster, representing another 28.02% of the pangenome and 6,510 gene clusters, 46.3% of the pangenome shared by some but not all isolate genomes. In addition, we highlight unique gene clusters identified within the isolated Baltimore genomes. Subsequent functional enrichment analysis revealed the presence of antimicrobial resistance (AMR) genes exclusive to this Baltimore. The unique AMRs are OXA-18, arr-2, dfrA12, and rmtF.

6 HORIZONTAL GENE TRANSFER ANALYSIS

6.1 Phylogenetic Trees

We first extracted the core genes from the 12 strains and constructed the phylogenetic tree (see Figure 2a). In this figure, we observe two main branches: one corresponding to *Escherichia coli* and the other to *Klebsiella pneumoniae*.

In Figure 2b, we selected nine genes, which are present in all 12 strains and belong to a plasmid. These genes are listed in Table S1. We then constructed a phylogenetic tree based on these genes.

PGF_07717077	PGF_00013945	PGF_00013871
PGF_04419840	PGF_00467825	PGF_00013893
PGF_00013875	PGF_01246464	PGF_00013866

Table S1. Selected genes present in all 12 strains and associated with a plasmid.

In this tree, we observe that the *E. coli* strains EC00701 and EC00678 are more closely related to KLP0015, a *Klebsiella* strain. This result aligns with expectations, as it has been demonstrated that horizontal gene transfer (HGT) of antibiotic resistance genes, belonging to the IncF plasmid, has occurred between these strains.

6.2 Clustering Based on Gene Presence and Absence

We performed a clustering analysis using the presence and absence of unique core genes across the 12 strains, as shown in Figure 3a. In this figure, *Klebsiella* strains are marked in pink, while *Escherichia* strains are marked in green. The phylogenetic tree clearly shows the separation of these groups.

Next, we focused on the family of genes present in the IncF plasmid and selected those found in all strains. We then generated another tree, as shown in Figure 3b. In this figure, we again observe the separation of *Klebsiella* and *Escherichia* groups. However, we did not detect horizontal gene transfer between them. It is worth mentioning that this presence/absence method, when used for tree comparison, is not effective in detecting horizontal gene transfer. Nevertheless, these data were later analyzed using persistent homology, which successfully identified horizontal gene transfer events.

6.3 HGT Detection

Finally, we used the program **HGT Detection 3.4** from T-REX Boc et al. (2012). This program computes a unique scenario of horizontal gene transfers (HGT) based on a given set of species and gene phylogenetic trees.

We first provided the program HGT Detection with the phylogenetic trees obtained from the core gene sequences and some genes from the IncF plasmid (Figure 2a, 2b). The program detected multiple horizontal gene transfer events, including one between EC00678 and KLP00715, as shown in Figure 4a.

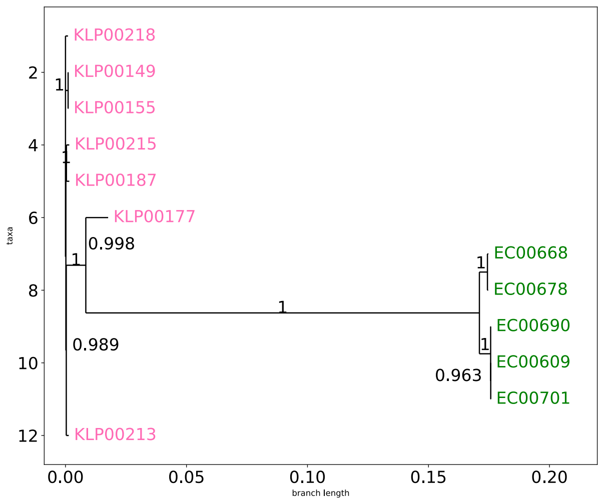

Figure 2a

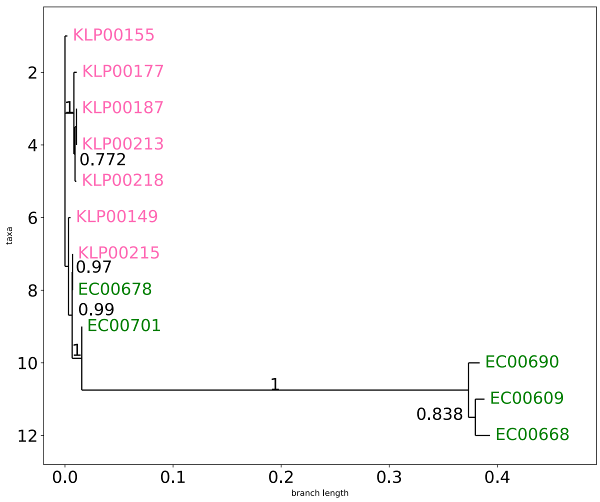

Figure 2b

Figure 2: Phylogenetic trees of 12 *Klebsiella* and *Escherichia* strains. *Klebsiella* strains are shown in pink, while *Escherichia* strains are shown in green. In Figure 2a, the phylogenetic tree was constructed using amino acid sequences of core genes. In Figure 2b, the phylogenetic tree was built using genes from the IncF plasmid listed in Table S1.

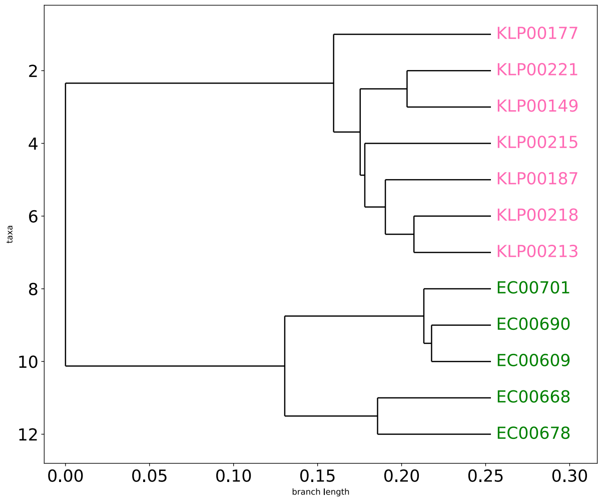

Figure 3a

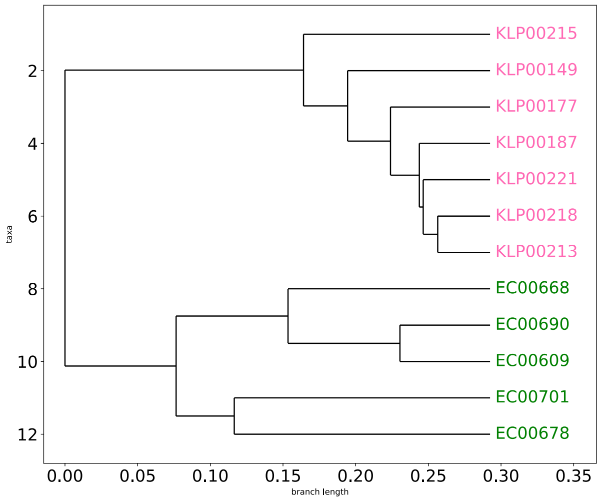

Figure 3b

Figure 3: Clustering results for core and plasmid genes. *Klebsiella* strains are shown in pink, while *Escherichia* strains are shown in green. In Figure 3a, clustering was performed using the count tables of core genes. In Figure 3b, clustering was based on the count tables of genes from the IncF plasmid

Similarly, we ran the program HGT Detection using the trees constructed from gene presence/absence data (Figure 3a, 3b). In this case, the program detected some horizontal transfer events, but only within the same groups of *Klebsiella* and separately within the group of *Escherichia* (Figure 4b).

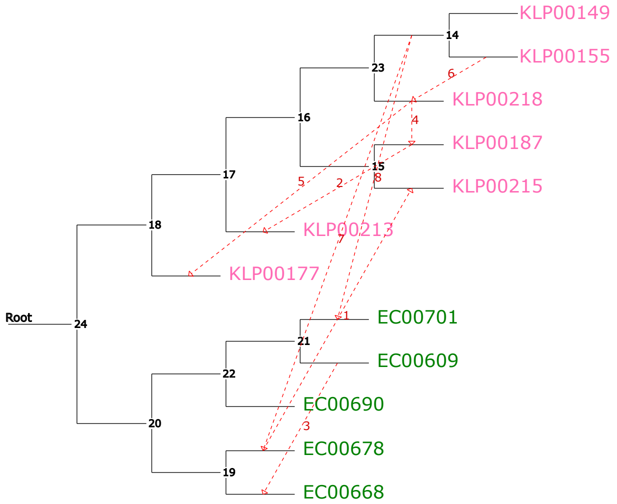

Figure 4a

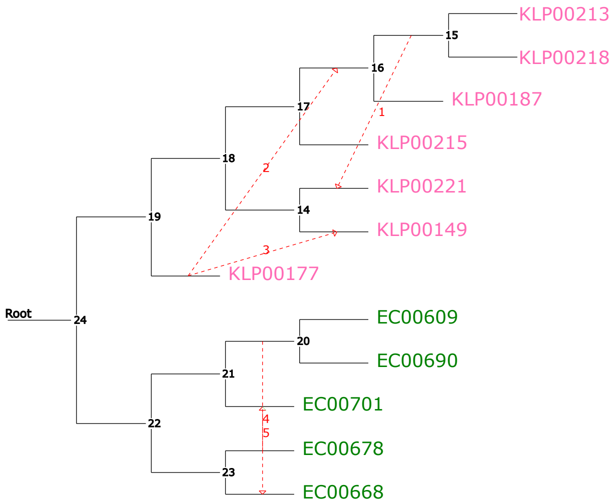

Figure 4b

Figure 4: Phylogenetic trees generated using HGT Detection 3.4. *Klebsiella* strains are shown in pink, while *Escherichia* strains are shown in green. Horizontal gene transfer (HGT) events detected by the software are highlighted in red. In Figure 4a, the species and gene trees were obtained from Figure 2, based on amino acid sequences. In Figure 4b, the species and gene trees were derived from Figure 3, using the gene presence/absence matrix

6.4 Interpretation of HGT Detection Results

The program iteratively detected HGT events and recalculated the fit criteria at each step:

- **Robinson and Foulds distance (RF)**: Measures the topological difference between the species and gene trees. Initially, RF was 20, indicating a significant difference.
- **Least-squares coefficient (LS)**: Represents the fitting of gene trees to the species tree. A high LS value suggests poor fit, which improved throughout the iterations.
- **Bipartition dissimilarity (BD)**: Quantifies the dissimilarity between bipartitions in the trees, starting at 21.5.

The program detected a total of **eight HGT events**, and after the last iteration (six), the metrics showed a perfect reconciliation between the gene and species trees:

- **Final RF = 0**, meaning complete topological agreement.
- **Final LS = 0.000**, indicating no residual fitting error.
- **Final BD = 0.0**, showing full concordance in bipartitions.

One of the most notable findings occurred during **Iteration 1**, where an HGT event was detected between **EC00678 and KLP00215**. These strains belong to *Escherichia* and *Klebsiella*, respectively, representing a cross-species horizontal gene transfer. This event suggests a potential genetic exchange between these two distinct bacterial genera, which could have significant implications for gene flow and adaptation.

These results confirm that the observed differences between species and gene trees can be fully explained by horizontal gene transfers.

REFERENCES

Alcock, B. P., Huynh, W., Chalil, R., Smith, K. W., Raphenya, A., Wlodarski, M. A., et al. (2022). CARD 2023: expanded curation, support for machine learning, and resistome prediction at the Comprehensive Antibiotic Resistance Database. *Nucleic Acids Research* 51, D690–D699. doi:10.1093/nar/gkac920

Altschul, S. F., Gish, W., Miller, W., Myers, E. W., and Lipman, D. J. (1990). Basic local alignment search tool 215, 403–410. Publisher: Elsevier

Boc, A., Diallo, A. B., and Makarenkov, V. (2012). T-rex: a web server for inferring, validating and visualizing phylogenetic trees and networks. *Nucleic acids research* 40, W573–W579

Delmont, T. O. and Eren, A. M. (2018). Linking pangenomes and metagenomes: the prochlorococcus metapangenome 6, e4320. doi:10.7717/peerj.4320. Publisher: PeerJ Inc.

Dey, T. K. and Wang, Y. (2022). *Computational topology for data analysis* (Cambridge University Press)

Edelsbrunner, H. and Harer, J. L. (2022). *Computational topology: an introduction* (American Mathematical Society)

El-Gebali, S., Mistry, J., Bateman, A., Eddy, S. R., Luciani, A., Potter, S. C., et al. (2019). The pfam protein families database in 2019 47, D427–D432. Publisher: Oxford University Press

Eren, A. M., Esen, C., Quince, C., Vineis, J. H., Morrison, H. G., Sogin, M. L., et al. (2015). Anvi'o: an advanced analysis and visualization platform for 'omics data 3, e1319. Publisher: PeerJ Inc.

Haft, D. H., Loftus, B. J., Richardson, D. L., Yang, F., Eisen, J. A., Paulsen, I. T., et al. (2001). TIGRFAMs: a protein family resource for the functional identification of proteins 29, 41–43. Publisher: Oxford University Press

Hyatt, D., Chen, G.-L., LoCascio, P. F., Land, M. L., Larimer, F. W., and Hauser, L. J. (2010). Prodigal: prokaryotic gene recognition and translation initiation site identification 11, 1–11. Publisher: Springer

Jones, P., Binns, D., Chang, H.-Y., Fraser, M., Li, W., McAnulla, C., et al. (2014). InterProScan 5: genome-scale protein function classification 30, 1236–1240. doi:10.1093/bioinformatics/btu031

Lees, J., Yeats, C., Perkins, J., Sillitoe, I., Rentzsch, R., Dessailly, B. H., et al. (2012). Gene3d: a domain-based resource for comparative genomics, functional annotation and protein network analysis 40, D465–D471. doi:10.1093/nar/gkr1181

Lu, J., Rincon, N., Wood, D. E., Breitwieser, F. P., Pockrandt, C., Langmead, B., et al. (2022). Metagenome analysis using the kraken software suite. *Nature Protocols* 17, 2815–2839. doi:10.1038/s41596-022-00738-y

Maria, C., Boissonnat, J.-D., Glisse, M., and Yvinec, M. (2014). The gudhi library: Simplicial complexes and persistent homology , 167–174

Rabadán R., B. A. (2020). *Topological data analysis for genomics and evolution*. doi:10.1017/9781316671665

Shaiber, A., Willis, A. D., Delmont, T. O., Roux, S., Chen, L.-X., Schmid, A. C., et al. (2020). Functional and genetic markers of niche partitioning among enigmatic members of the human oral microbiome 21, 292. doi:10.1186/s13059-020-02195-w

Utter, D. R., Borisy, G. G., Eren, A. M., Cavanaugh, C. M., and Mark Welch, J. L. (2020). Metapangenomics of the oral microbiome provides insights into habitat adaptation and cultivar diversity. *Genome Biology* 21, 293. doi:10.1186/s13059-020-02200-2

Van Dongen, S. and Abreu-Goodger, C. (2012). Using MCL to extract clusters from networks , 281–295Publisher: Springer

Wilson, D., Pethica, R., Zhou, Y., Talbot, C., Vogel, C., Madera, M., et al. (2009). SUPERFAMILY—comparative genomics, datamining and sophisticated visualisation 37, 14
